# The Role of IGF/IGF-1R Signaling in Hepatocellular Carcinomas: Stemness-Related Properties and Drug Resistance

**DOI:** 10.3390/ijms22041931

**Published:** 2021-02-16

**Authors:** Mai-Huong Thi Ngo, Han-Yin Jeng, Yung-Che Kuo, Josephine Diony Nanda, Ageng Brahmadhi, Thai-Yen Ling, Te-Sheng Chang, Yen-Hua Huang

**Affiliations:** 1 International PhD Program for Cell Therapy and Regeneration Medicine, College of Medicine, Taipei Medical University, Taipei 11031, Taiwan; ntmhuong@hpmu.edu.vn (M.-H.T.N.); fine.nanda@gmail.com (J.D.N.); brahmadhi@gmail.com (A.B.); 2 Department of Biochemistry and Molecular Cell Biology, School of Medicine, College of Medicine, Taipei Medical University, Taipei 11031, Taiwan; 3 Research Center of Cell Therapy and Regeneration Medicine, Taipei Medical University, Taipei 11031, Taiwan; m609108001@tmu.edu.tw (H.-Y.J.); edditkuo@tmu.edu.tw (Y.-C.K.); 4 Department and Graduate Institute of Pharmacology, National Taiwan University, Taipei 11031, Taiwan; 5 School of Traditional Chinese Medicine, College of Medicine, Chang Gung University, Taoyuan 33382, Taiwan; 6 Division of Internal Medicine, Department of Gastroenterology and Hepatology, Chang Gung Memorial Hospital, Chiayi 61363, Taiwan; 7 Graduate Institute of Medical Sciences, College of Medicine, Taipei Medical University, Taipei 11031, Taiwan; 8 Center for Reproductive Medicine, Taipei Medical University Hospital, Taipei 11031, Taiwan; 9 Comprehensive Cancer Center, Taipei Medical University, Taipei 11031, Taiwan; 10 Research Center of Cancer Translational Medicine, Taipei Medical University, Taipei 11031, Taiwan; 11 PhD Program for Translational Medicine, College of Medical Science and Technology, Taipei Medical University, Taipei 11031, Taiwan

**Keywords:** insulin-like growth factor, liver cancer, cancer stemness, IGFs inhibitor, targeting drug resistance

## Abstract

Insulin-like Growth Factor (IGF)/IGF-1 Receptor (IGF-1R) signaling is known to regulate stem cell pluripotency and differentiation to trigger cell proliferation, organ development, and tissue regeneration during embryonic development. Unbalanced IGF/IGF-1R signaling can promote cancer cell proliferation and activate cancer reprogramming in tumor tissues, especially in the liver. Hepatocellular carcinoma (HCC) is one of the leading causes of cancer-related death, with a high incidence and mortality rate in Asia. Most patients with advanced HCC develop tyrosine kinase inhibitor (TKI)-refractoriness after receiving TKI treatment. Dysregulation of IGF/IGF-1R signaling in HCC may activate expression of cancer stemness that leads to TKI refractoriness and tumor recurrence. In this review, we summarize the evidence for dysregulated IGF/IGF-1R signaling especially in hepatitis B virus (HBV)-associated HCC. The regulation of cancer stemness expression and drug resistance will be highlighted. Current clinical treatments and potential therapies targeting IGF/IGF-1R signaling for the treatment of HCC will be discussed.

## 1. Introduction

Hepatocellular carcinoma (HCC) accounts for about 75–85% of all primary liver cancer [1,2], which is one of the top five causes of cancer deaths worldwide [2]. HCC can develop from loss of cell cycle control in adult hepatocytes or progenitor cells. Several risk factors for developing HCC have been identified, including hepatitis virus infection, abnormal fatty acid metabolism, alcoholic liver disease, and toxins [1,3,4]. The dominant cause varies across different geographical areas [5].

In five main types of hepatitis virus (type A, B, C, D, and E), types B and C cause the most public health burden, as they are responsible for more than half number of HCC [6]. Moreover, according to an investigation on the global burden of viral hepatitis from 1990 to 2013, about 96% of the viral hepatitis-related mortality are caused by hepatitis B and C [7]. In 2015, around 257 million people had chronic hepatitis B virus (HBV) infection, and 71 million had hepatitis C virus (HCV) infection [8]. A goal of the World Health Organization’s Global Strategy for Viral Hepatitis Elimination in 2016 was to reduce hepatitis-related deaths by 65% by 2030 (from 1.34 million deaths annually to less than 0.5 million) [9].

Therapeutic strategies available for HCC, such as resection, transplantation, ablation, radiotherapy, chemotherapy, and molecular targeting therapy, are highly dependent on the stage of liver cirrhosis and on the offerings of the medical center, which varies among different countries [1,3,4,10]. Targeted therapies (sorafenib and lenvatinib) have been approved for patients with advanced HCC who are not eligible for local treatments. However, the median overall survival (OS) with either sorafenib or lenvatinib is only about 13 months [11,12,13]. Therefore, an improvement for targeted therapies is needed.

Recently, Insulin-like Growth Factor (IGF)/IGF-1R signaling has become a potential target for HCC treatment [14,15]. Elevation in the activation of IGF/IGF-1R signaling in patients with liver cancer caused by hepatitis B infection has been widely reported. In this article, we summarize the abnormal activity of IGF/IGF-1R signaling in HCC, the limitations of current targeted therapies for advanced HCC, and drugs that target IGF/IGF-1R signaling for the treatment of specific HCC.

## 2. The Etiology of HCC

### 2.1. Virus Infections Initiate HCC

HCC is often associated with HBV and HCV. According to a 2016 report by the U.S. Centers for Disease Control and Prevention, approximately 65% of liver cancers are associated with the hepatitis B or C virus. In a study of 3843 patients in Taiwan, hepatitis viruses were associated with more than 80% of HCC [6].

HBV is a small DNA virus with a partially double-stranded DNA of 3200 bp [16]. HBV infection is responsible for 66% of virus-caused HCC deaths worldwide [8]. The highest prevalence of HBV infection is reported in the African (6.1%) and Western Pacific (6.2%) regions [8]. Most cases of HBV are transmitted through bodily fluids, such as via blood transfusion, sexual contact, or from mother to child. The HBV virus can amplify independently inside hepatocytes; integration of HBV into the hepatocyte genome can increase carcinogenic opportunities in HBV-infected patients.

The HBx gene is mainly responsible for HBV-associated HCC development. The HBV DNA contains four open reading frames (ORFs), which code for surface antigen (S) protein, precore (C) protein, polymerase (P) protein, and X proteins [17,18]. The ORF-X is the smallest ORF with 462 bp, and it acts as a viral production promotor within the cell [19,20]. Silencing the X gene results in the suppression of HBeAg production and viral production [21,22]. HBx exerts its effects on cell cycle progression and the normal physiology of hepatocytes by upregulating levels of G1 proteins [23]. In addition, HBx initiates HCC development by upregulating Ras/Raf/MAPK signaling, PI-3K/Akt signaling, Jak/STAT signaling, and NFκB signaling [18,24].

HBV perinatal transmission is more effective because the immune systems have not fully matured yet in fetus [25]. Acquisition of HBV in the early life causes chronic infection in most cases while infection in adults is usually recovered with subsequent acquired immunity [26,27,28]. A study of 1280 seronegative patients from 12 Yupik Eskimo villages in America demonstrated that the rate of chronic hepatitis B in HBV infected patients declined with increasing age. Percentages for the ≤4 years group, 5–9 years group, and adults (>30 years) group were 28%, 16.4%, and 7.7%, respectively [27]. Fortunately, there is a vaccine to prevent HBV infection that is effective for all ages including infants, children, and adults [8].

The HCV pandemic affects all regions worldwide; the highest prevalence occurred in Central Asia, East Europe, and central and Western Saharan of Africa [8,29]. Globally, there were 1.75 million new HCV infections and 57 million people living with chronic HCV in 2015 [8]. Approximately, 75% of patients with acute HCV infection progress to develop chronic HCV, and the risk of developing cirrhosis/HCC from chronic HCV is approximately 10–20% [30,31].

Unlike HBV, HCV is an RNA virus, which makes it difficult to insert into the hepatocyte genome. Therefore, its carcinogenic activity is linked to indirect mechanisms. The HCV relies on endoplasmic reticulum (ER) in the hepatocyte to produce viral proteins, thus causing the ER stress. The ER is an important organelle that helps to maintain normal functions of hepatocytes, such as the transportation of proteins and lipids and the synthesis of proteins [32,33,34]. As a result, injuring hepatocytes by HCV could lead to cirrhosis.

There is no effective HCV vaccine; however, effective HCV therapies are available and work well. Direct-acting antivirus (DAA) therapy was introduced in 2013 and became recommended first line treatment for HCV by WHO guidelines in 2014 [8]. DAA therapy can cure 95% of HCV infections [31].

Coinfection with both virus types raises the risk for cirrhosis progression [35,36], and cirrhosis progression is highly possible to lead to HCC initiation. Coinfected patients show a higher rate of cirrhosis than HBV mono-infected patients (44% vs. 21%) [37]. In addition, the percentage of cirrhosis and HCC in dual-infected patients is higher than in HCV mono-infected patients (95% vs. 48.5 and 63% vs. 15%, respectively) [38]. Coinfected patients were more often immigrants from Africa and Asia than HCV- or HBV-mono-infected patients (52% vs. 20% and 22%, respectively, *p* = 0.01) [36]. In addition, 2.7 million patients are coinfected with HBV-HIV and 2.3 million patients are coinfected with HCV-HIV [8]. Coinfection with HBV/HCV and HIV raises the risk of liver cirrhosis and HCC development [39,40,41]. The liver-related mortality rate of patients with HBV-HIV (14.2/1000) is higher than that of patients with only HIV (1.7/1000) or only HBV (0.8/1000) [39].

### 2.2. Obesity and NAFLD Cause HCC

Obesity is rapidly becoming a health problem all over the world, especially in Western countries. It is established that more than 2 billion people are overweight or obese worldwide. By the year 2030, it is projected that 38% of adults will be overweight and 20% will be obese if this trend is not changed [42]. It is well known that obesity is highly associated with other health problems such as cardiovascular disease, stroke, hypertension, and cancer. A meta-analysis of data from 1,779,471 individuals from articles published from 1996 to 2011 found a positive correlation between body mass index (BMI) and risk of liver cancer. Persons with a BMI of 25, 30, or 35 kg/m^2^ had a 1.02, 1.35, or 2.22 fold relative risk of liver cancer, respectively [43]. In a retrospective analysis of 714 patients with HCC who underwent curative hepatectomy, the 5-year OS rate of HBV-HCC patients with BMI ≥ 25 kg/m^2^ (65%) was lower than that of HBV-HCC patients with BMI < 25 kg/m^2^ (85%). However, among patients with HCV-HCC, those with BMI ≥ 25 kg/m^2^ had a better 5-year OS rate than those with BMI < 25 kg/m^2^ (75% vs. 65%) [44].

Recently, nonalcoholic steatohepatitis (NAFLD), which is caused by obesity and some hepatic histological damage, became the major cause of chronic liver disease in Western countries [45]. The risk of HCC developing in nonalcoholic steatohepatitis (NASH)-associated cirrhosis was 2.4–12.8% while that of HCC developing in NASH without cirrhosis was low (0–3%) [46]. Besides, a study, which compared 296,707 patients with NAFLD with 296,707 matched control, showed that the HCC incidence was significantly higher among NAFLD patients versus control (0.02/1000 person-years; hazard ratio, 7.62, 95% confidence interval = 5.76–10.09) [47]. Similarly, a data analysis from four databases which included 18,782,281 eligible individuals from United Kingdom, Netherlands, Italy, and Spain showed that patients with NAFLD/NASH had cirrhosis risk and HCC risk significantly higher than controls with pooled hazard ratios 4.73 (95% CI 2.43–9.19) and 3.51 (95% CI 1.72–7.16), respectively [48]. The data of 25,947 subjects in Korea from September 1, 2004, to December 31, 2005, indicated the NAFLD was associated with the development of HCC. The cancer incidence rate of patients with NAFLD was significantly higher than that of control (782.9 version 592.8/100,000 person-years; hazard ratio 1.32; 95%CI 2.09–133.85; *p* < 0.001) [49].

Furthermore, the risk of NAFLD-related HCC increased quickly in the last two decades. A study that included 323 HCC patients from 1995–1999 to 2010–2014, indicated that the prevalence of NAFLD-HCC increased from 2.6% to 19.5%, respectively, *p* = 0.003 [50]. In addition, among 158,347 adult liver transplant candidates in United State, the proportion of patients with HCC increased from 6.4% (2002) to 23% (2016) (trend *p* < 0.001) [51].

Together, these data suggest that the risk of HCC due to NAFLD is going more serious while that of HCC due to HCV/HBV infection is going better of control. However, until recently, there is no consensus on optimal HCC screening measures for patients with NAFLD/NASH.

### 2.3. Other Factors That Cause HCC

Aflatoxins, a group of mycotoxins produced by the fungi *Aspergillus flavus* and *Aspergillus parasiticus*, account for a large part of toxin-related HCC. People in tropical countries may ingest aflatoxin through fungal-contaminated food that was improperly stored in high humidity and temperature. Aflatoxin causes an arginine–to-serine mutation at codon 249 of the p53 gene, leading to cancellation of the tumor suppression functions of this gene. A study using HCC samples from high and low risk areas of aflatoxin showed that the third nucleotide guanine to thymine transversion mutation at codon 249 was present in 57% and 10% of samples, respectively [52]. Similarly, liver cancer cell lines that were induced to express high levels of CYP450 were more sensitive to the cytotoxic effect of aflatoxin than parental cells. The third nucleotide guanine to thymine transversions in the codon number 249 and the first nucleotide cytosine to adenine transversions at codon number 250 of p53 gene were found at a high frequency [53]. A high incidence of HCC is found in areas where aflatoxin and HBV infection are common, raising speculation regarding a synergic carcinogenic interaction between aflatoxin and HBV infection [54]. A study on HCC samples from Guangxi, China, confirmed the positive correlation between HCC and aflatoxin but evidence for an HBV-aflatoxin interaction modulating the p53 mutation [55] or for a hepatitis B surface antigen (HBsAg) and aflatoxin synergistic effect in HCC [56] was not clear.

Alcohol consumption, another risk factor for HCC, induces liver cancer development through steatosis, steatohepatitis, and cirrhosis. The acetaldehyde and lipid peroxidation from ethanol metabolism in the liver creates protein adducts and DNA adducts, which can trigger liver injury and fibrogenesis [57,58,59]. In addition, alcohol metabolism-derived ROS can destroy large organelles and alter the structure and function of DNA [60]. Oxidative stress in the liver promotes the secretion of cytokines and chemokines (IL-6, IL10, IL1β, and TNFα) [61,62,63,64] and activates several signaling pathways (Jak/STAT; NF-kB, and MAPK cascade) which are implicated in the initiation of HCC. Findings from a systematic review and meta-analysis indicate that the relative risks of liver cancer for moderate drinking (<3 drinks/day) and heavy drinking (≥3 drinks/day) are 0.91 and 1.16, in comparison to non-drinking. The study also found an estimated excess risk of 46% or 66% in drinkers who consumed 50 g or 100 g alcohol per day [65]. A projected prevalence study has predicted that, if current trends continue, deaths due to alcohol-related liver disease will increase from 8.23 deaths/100,000 person-years in 2019 to 15.20 deaths/100,000 person-years in 2040 [66].

## 3. IGF/IGF-1 Signaling in HCC

### 3.1. Abnormal IGF/IGF-1 Signaling in HCC

#### 3.1.1. Increases in IGF-1/IGF-2 Secretion

While it is still unclear whether IGF-1/IGF-2 secretion increases or decreases in HCC, studies of HBV-HCC have shown an increase in IGF-1/IGF-2 secretion [67,68,69,70]. IGF-1 and IGF-2 secretion is mediated by growth hormone (GH), which is released from anterior pituitary cells and was found to directly stimulate the transcription of IGFs in hepatocytes, the major source of IGF-1 and IGF-2, in mice and rats [71,72]. After receiving the stimulating signal from GH, hepatocytes first synthesize the precursor of IGF-1, which is later cleaved to produce mature IGF-1 peptide [73]. Several studies have reported an increase in IGF-1 levels in the serum of prostate, breast, and colon cancers [74]. Moreover, in patients with acromegaly, a high level of GH is believed to increase the level of circulating IGF-1 [75].

In contrast, in patients with liver cirrhosis, the low-affinity of the GH receptor in the liver could induce the resistance of GH stimulation and decrease IGF-1 secretion [76]. It has been reported that the level of IGF-1 in the plasma of healthy 61 to 75 year old individuals ranges from 61 to 210 μg/mL [77], while it ranges from 27.7 to 68.6 μg/mL in patients with HCC [78]. Thereafter, the low levels of IGF-1 would lead to an increase in GH production by positive feedback [74]. Interestingly, Cao et al. measured IGF mRNA and protein levels in patients with HCC after partial hepatectomy, and found that both levels were significantly increased in patients who received treatment with recombinant human (rhGH) compared with patients who did not receive rhGH treatment, suggesting that rhGH has the capability to improve the GH/IGF-1 axis after partial hepatectomy in HCC patients [79]. IGF-2 secretion appears to be less dependent on the stimulation of GH, compared to IGF-1 [80,81].

The upregulation of IGF-2 expression in HCC may be regulated by epigenetic mechanisms. An integrative oncogenomic analysis to elucidate the mechanisms of IGF-2 overexpression and its oncogenic activities in HCC found that a pro-proliferative receptor with a high affinity for IGF-2, INSR-A isoform, was upregulated in HCC samples. Several microRNAs (miRNAs) were also upregulated, including miR-483-5p, which has been found to increase IGF-2 levels [82]. Furthermore, an aberrant methylation pattern on the IGF-2 promotor was identified in the samples with high IGF-2 expression. The IGF-2-high expressing samples showed demethylation on the fetal promotor but increased methylation on the adult promotor [83].

An activated IGF/IGF-1R signaling pathway is associated with activation of the Ras–MAPK pathway and PI 3-Kinase pathway. These pathways activate variable bioactivities, such as cell growth, cell differentiation, cell survival, and protein synthesis [84]. An increased level of IGF-2 is observed in patients with HCC. IGF-2 also binds to IGF-1R and may lead to cancer cell proliferation in HCC patients. A study by Martinez-Quetglas et al. evaluated levels of IGF-2 mRNA and protein in patients with HCC and found that 15% of human HCC tissues had IGF-2 levels 20 times greater than levels in non-tumor liver tissues, and activation of the IGF-2 signaling pathway increased the expressions of AKT1 and MYC [83]. In diethylnitrosamine (DENA)-induced hepatocellular carcinoma rat models, IGF-2 levels were higher in rat liver tumors than in normal rat liver. Moreover, IGF-2 might promote proliferation through a paracrine mechanism in the early stage of carcinogenesis. After transformation, the malignant cells were able to secrete IGF-2 and induce proliferation by an autocrine mechanism [85].

Notably, levels of IGFs are not the only factor that contributes to IGF activity. Although research regarding the effects of abnormal posttranslational modifications of IGF-1 peptide precursor in HCC are scarce, posttranslational modification of IGF-1 peptide precursor has been reported to contribute to the activity of IGF-1 [86].

Interestingly, while the liver is the predominant IGF-1 producing organ under normal conditions, it lacks IGF receptors. However, IGF receptors have been found in damaged liver, suggesting that damaged livers may serve as targets for IGF-1 in order to maintain liver homeostasis and architecture. In a partial IGF-1 deficiency murine model, the liver was induced to express IGF-1 receptors along with elevated acute phase and inflammatory proteins, which resulted in liver oxidative damage. The IGF-1 partial deficiency was also associated with altered expression of genes involved in the cytoskeleton, extracellular matrix, cell junctions, and hepatocyte polarization [87].

#### 3.1.2. Increases in IGF-1R Expression

IGF-1R is the receptor for both IGF-1 and IGF-2, and several oncogenes targeting IGF-1R transcription have been reported to cause the overexpression of IGF-1R in cancers. For example, Reiss and co-workers observed that when the proto-oncogene c-myb is constitutively expressed in mouse fibroblasts, the requirement for adding extra IGF-1 in the culture medium while culturing wildtype fibroblasts is no longer necessary, implying that an increase of IGF-1 secretion occurred in the cells expressing c-myb. In addition, IGF-1R mRNA levels were upregulated in the cells expressing c-myb compared with the control cells, suggesting that the presence of c-myb can positively regulate IGF-1 secretion [88].

Likewise, the loss-of-function of certain tumor suppressors can also increase IGF-1R expression. The tumor suppressor p53 is able to suppress transcription of IGF-1R, thereby decreasing the expression of IGF-1R. Results of co-transfecting wildtype or mutant p53 together with the IGF-1R promotor in an osteosarcoma cell line lacking endogenous p53 suggest that wildtype p53 suppresses the IGF-1R promotor while mutant p53 does not [89]. Thus, oncogenes and mutated tumor suppressors that target IGF-1R transcription appear to cause dysregulation of IGF-1R expression.

Overexpression of IGF-1R is frequently observed in tumors and is a marker for poor prognosis in HCC patients [90]. IGF/IGF-1R signaling mediates cell proliferation, cell survival, migration, and protein synthesis and can block apoptosis by expressing Myc and Akt1 in the liver, thereby causing invasion and metastasis of tumor cells [91]. Thus, IGF-1R and the molecules that participate in the IGF/IGF-1R signaling pathway have become targets for HCC therapies. Xentuzumab is a monoclonal antibody against IGF-1 and IGF-2. In 2016, Martinez-Quetglas et al. studied the efficacy of xentuzumab on inhibiting the IGF signaling pathway and the drug’s effects on HCC and found that xentuzumab, with or without sorafenib, inhibited IGF-1R phosphorylation as well as AKT signaling in the liver of mice with xenograft HCC tumors. Xentuzumab also exhibited efficacy in suppressing tumor growth and improving the survival rate of the mice. Moreover, decreases in cell proliferation and colony formation were identified in liver cancer cells treated with xentuzumab [83].

#### 3.1.3. Reduction in IGFBP Secretion

Insulin-like growth factor binding proteins (IGFBPs) are secreted proteins that modulate IGF/IGF-1R signaling. They bind to IGF-1 or IGF-2 and prevent activation of IGF/IGF-1R signaling. The affinity of IGFBP to IGF-1/IGF-2 is higher than that of IGF-1/IGF-2 to IGF receptors therefore, once IGFBP-IGF-1/IGF-2 is formed, the IGF/IGF-1R signal is blocked [92,93]. Studies of IGF and IGFBP levels in blood circulation show that most circulating IGF remains as a complex with IGFBPs and only 1% of total IGF-1 in blood circulation exists as free IGFs [94]. The level of circulating IGFBPs in the plasma of patients with HCC was different than that from the plasma of healthy donors; however, studies agree that reduction of IGFBP3 and IGFBP7 promotes HCC [95,96,97,98,99].

IGFBP3 is the most common of the IGFBPs, accounting for 90% of the total IGFBPs in serum [95,100]. Studies have found that IGFBP3 is downregulated in HCC [92,96], and this is associated with a poor prognosis [101]. IGFBP3 is also downregulated in the majority of hepatoblastoma primary tumors [97] and is implicated in HCC drug resistance [102]. IGFBP3 regulates cell growth though the TGF-β or Rb pathways [96], promotes DNA methylation [97], and modulates apoptosis and the cell cycle [103]. Compared with either IGF-1 or IGF-2, IGFBP3 has been found to be a more effective predictor of HCC development in patients with chronic HCV infection [104].

IGFBP3 secretion is decreased in HCC patients, and this is associated with changes in IGFBP3 transcription. It was found that IGFBP3 is expressed at low levels in a hepatoblastoma (HB) cell line and in HB primary tumors while it is expressed at high levels in normal liver tissues. In addition, a methylation analysis of CpG sites revealed that hypermethylation occurs on the promotor region of IGFBP3 in metastatic HB cells [97]. Another study found that miR-155, an oncogene-associated miR that is frequently upregulated in HCC, may be responsible for decreasing IGFBP3 expression. An increase of miR-155, which is seen in liver tissues of patients with HCV-derived HCC and in HCC cell lines, can cause IGFBP3 expression to decrease, while transfection of miR-155 inhibitor into HCC cells can rescue IGFBP3 expression [105]. Moreover, the downregulation of IGFBP3 can be mediated by the binding of T-cell-restricted intracellular antigen-1 (TIA-1) to its AT-rich element in the 3′-untranslated region [106].

The downregulation of IGFBP7 has also been correlated with poor prognosis in HCC. Reduced expression of IGFBP7 was observed in a quarter of HCC patients and in HCC cell lines and may be due to increased methylation of the IGFBP7 promotor [107], as IGFBP7 has a high-affinity to bind IGF-1, blocking IGF/IGF-1R signaling. Overexpression of IGFBP7 suppressed cell growth, tube formation, tumor development, and neovascularization in a chicken embryo [107]. Additionally, deleting IGFBP7 promoted HCC development in a mouse model [99]. Taken together these results suggest that IGFBP7 may have potential to predict progression in HCC.

#### 3.1.4. Increases in IGFBP Protease Secretion

HCC contain high levels of IGFBP proteases, which may lead to activation of IGF/IGF-1R signaling. While no IGFBP protease activity was detected in the conditioned medium from normal rat livers [108], an increase in IGFBP protease activity was observed in HCC cells [109]. IGFBP proteases are known to cleave IGFBPs specifically into small fragments, thereby releasing IGFs due to the reduced affinity of the IGFBP-IGF complex and activating IGF-mediated signaling that leads to cell proliferation, cell migration, tumor growth, and angiogenesis in several cancers, including HCC. [110,111,112].

IGFBP proteases can be separated into three groups: kallikrein-like serine proteases (such as prostate-specific antigen (PSA), and *γ*-nerve growth factor (*γ*-NGF)), cathepsins (such as Cathepsin D), and matrix metalloproteinases (MMPs) [110,113,114]. Kallikrein has been proposed to be a useful biomarker in cancer [115]. According to RNA sequencing gene expression data from The Cancer Genome Atlas (TCGA), genes for several types of kallikrein were significantly associated with HCC [116]. Moreover, compared with healthy controls, the concentration of plasma Cathepsin D was as much as 10 times higher in patients with various liver diseases such as cirrhosis, primary hepatoma, hepatitis infection, or hepatocarcinoma [117]. Another study evaluating the potential value of cathepsins (cathepsin D, B, and L) as markers for malignant progression in patients found that the serum levels of all cathepsins (D, B, and L) were increased in patients with HCC and elevated levels of two cathepsins (cathepsin D and B) were seen in patients with cirrhosis [109]. Furthermore, Yeh et al. evaluated the utility of MMP-9/MMP-2 ratios as a biomarker in HCC. The ratio was found to be significantly different in patients with different stages of HCC, with higher MMP-9/MMP-2 ratios seen in patients with advanced HCC as compared to those in early-stage HCC. The authors propose that the ratio not only serves as a marker distinguishing healthy individuals from HCC patients but also helps to discriminate advanced from early-stage HCC [118]. Together, these studies support the use of IGFBP proteases as potential biomarkers for detecting precancerous liver disease [109,117].

#### 3.1.5. Virus Infection Promotes Dysregulation of IGF/IGF-1R Signaling in HCC

Hepatitis virus infection can stimulate IGF signaling, increasing the likelihood of HCC progression. HBV is highly associated with HCC formation and has been reported to involve IGF/IGF-1R signaling in HCC. A study on the human HBV transactivator X-gene product (HBV-X) demonstrated that protein X expressed by human HBV can promote IGF-1 binding and increase the expression of IGF-1 receptors [119]. HBV-X was also shown to upregulate endogenous IGF-2 transcription via Sp1 binding sites on IGF-2 promotor 4 [120]. These results suggest that that HBV-X induces IGF-2 expression and signaling, hence contributing to the development of HCC.

The correlation between virus infection and IGF signaling was also identified in HCC patients with HCV infection. In 2008, Sohda et al. reported immunohistochemical evidence of IGF-2 in human small HCC tissues with HCV infection, while no IGF-2 signal was found in normal hepatocytes. Moreover, the more changes in fatty acids metabolism in HCC tissues, the more IGF-2 immunoreaction was detected. It was suggested that since insulin can regulate the metabolism of fatty acids, changes in IGF-2 levels may be associated with both HCC proliferation and fat metabolism [121].

#### 3.1.6. Microenvironment Regulates IGF/IGF-1R Signaling in HCC

**Figure 1:**
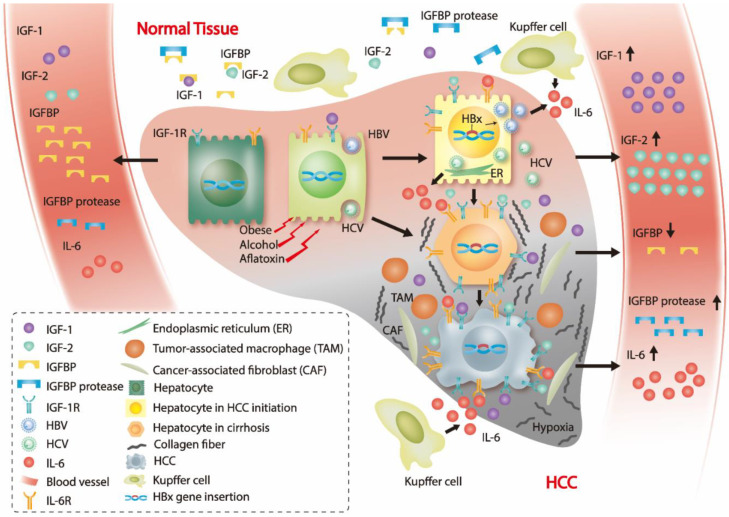
The insulin-like growth factor (IGF)/IGF-1R signaling pathway in the development of liver disease. During the initiation of liver diseases caused by hepatitis virus infection, obesity, alcohol, or toxins, hepatocytes increase the release of inflammatory cytokines, such as IL -6. IL-6 can also be secreted by Kupffer cells after hepatitis B virus (HBV) infection, activating the IGF/IGF-1R signaling pathway. Released cytokines recruit TAMs and CAFs to the hepatocytes, leading to cirrhosis formation. IGF/IGF-1R signaling is activated following disease progression. Meanwhile, the levels of IGF-1, IGF-2, Insulin-like growth factor binding proteins (IGFBPs), and IGFBP proteases can change. Cirrhosis in the liver may develop into hepatocellular carcinoma (HCC). In HCC, more TAMs and CAFs are recruited to the cancer microenvironment and hypoxia occurs in the liver. Furthermore, the expression of IGF-1, IGF-2, IGF-1R, and IL-6 increase.

The tumor microenvironment contains numerous factors, such as cytokines, cancer-associated fibroblasts (CAFs), pericytes in the liver, tumor cells, macrophages, and endo/epithelial cells that can influence IGFs signaling in HCC (Figure 1). Since the IGF/IGF-1R signaling pathway is a target of cancer therapies, investigation into the associations between the tumor microenvironment and IGF/IGF-1R signaling may aid in the development of therapeutic strategies [122].

Studies of the correlations between HIF1α, HBx protein, and IGF/IGF-1R signaling have indicated that hypoxia is involved in IGF/IGF-1R activation in HCC. HBV-HCC patients expressed a higher level of IGF/IGF-1R signaling activity than non-HBV HCC patients [69]. Similarly, overexpressed HBx has been observed to upregulate HIF1α protein expression in vitro, and hypoxia can induce the accumulation of IGF-1R [123]. Together these results suggest that HIF1α may be useful as a prognostic factor in patients with HBV-HCC [124].

Mesenchymal stem cells (MSCs) can be recruited into the tumor microenvironment to regulate IGF signaling during the HCC development. Although the role of MSCs in the tumor microenvironment is still unclear, it has been shown that MSC secretions can downregulate IGF/IGF-1R signaling and inhibit HCC cell proliferation. In 2015, Yulyana et al. showed that conditioned media (CM) derived from human fetal MSCs (hfMSCs) expressed high levels of IGFBPs, which can bind free IGFs thereby decreasing their binding with IGF receptors. Moreover, studies with patient-derived orthotopic HCC mouse xenograft models showed that tumors from mice injected with CM derived from hfMSCs were significantly reduced compared with tumors injected with control medium or CM derived from MRC5 fibroblasts [125]. This suggests that CM derived from MSCs may be an alternative strategy for the treatment of HCC.

CAFs in the tumor microenvironment can regulate IGFs secretion and support the progression of cancer cells. For example, CAFs have been shown to express IGF-2 and to upregulate IGF/IGF-1R signaling in non-small cell lung cancers. This remodulation of IGF/IGF-1R signaling can induce Nanog expression and contribute to cancer stemness [126]. Notably, another finding has suggested that there are complex interactions between CAFs, tumor cells, and IGF/IGF-1R signaling. In colon cancers, TGFβ activation is induced in CAFs, which can contribute to the upregulation of IGFBP7, a TGFβ-induced gene product secreted by tumor cells. The IGFBP7 secreted by tumor cells can promote anchorage-independent growth and colony formation in malignant mesenchymal cells and in epithelial cells with an EMT phenotype. These findings suggest that the function of IGFBP7 can be completely different when it is secreted by tumor cells or by non-tumor cells [127].

### 3.2. IGF/IGF-1R Signaling Induces Expressions of Stemness-Related Proteins in HCC

#### 3.2.1. IGF/IGF-1R Signaling Induces Expression of Cancer Stemness-Related Transcription Factors

IGF/IGF-1R signaling has been reported to induce the expression of cancer stemness-related transcription factors, including IGF-1R, NANOG, OCT4, NFκB, STATs, SOX2, and YAP.

IGF-1R is well known not only as a location membrane protein but also as a nuclear translocation protein. IGF-1R directly binds to DNA or binds to other transcription factors to induce gene transcription [128,129]. The activation of the Wnt/β-catenin signaling pathway is associated with promoting self-renewal of liver cancer stem cells [130]. A study in HepG2 cells showed that overexpression of IGF-1R upregulates the transcriptional activity of β-catenin and cyclin D1 3.5-fold and 2.3-fold, respectively [131]. Bodzin et al. demonstrated that IGF-1R nuclear translocation increased in CD133-HCC Mahlavu cells treated with the cancer drug getifinib, in a dose-dependent manner [132].

NANOG and OCT4 are highly activated in a variety of stem cells including cancer stem cells. An association between high expression levels of NANOG/OCT4 and poor prognosis in HCC patients has been shown [133,134]. Shan et al. isolated NANOG-positive cancer cells and identified the co-expression of OCT4 and SOX2 genes with the presence of stemness characteristics (sphere formation, self-renewal, differentiation, and malignancy) in these cells. Noticeably, IGF/IGF-1R signaling was identified as the molecular mechanism involved in regulating the cancer stemness characteristics of these cells. Blocking IGF/IGF-1R signaling by picropodophyllin (PPP), an IGF-1R inhibitor, also inhibited the expression of NANOG and sphere formation ability in these cells [133]. The regulation of NANOG and OCT4 in HCC cancer stem cells by IGF/IGF-1R signaling was elucidated in HCC cell lines. IGF-1 treatment induced the expression of NANOG and OCT4 and sphere formation in HepG2.2.15, Hep3B, and PLC5 cell lines, and inhibition of IGF-1R by shIGF-1R suppressed cancer stemness properties in these cell lines [134].

NFκB is a well-known transcription factor that induces cell proliferation and self-renewal properties of cancer stem cells. IGFBP acts as a modulator of IGF-1-activity. The conditioned media of hfMSCs, which contains a high level of IGFBP, was found to suppress HCC cell proliferation and patient-derived cancer stem cell growth. These findings indicate that the tumor inhibiting activity of this medium was through disruption of IGF-1R-Akt-NFκB signaling [125].

The signal transducer and activator of transcription (STAT) protein family has seven members, STAT1-5, STAT5B, and STAT6 [135,136]. STAT3 has been found to be involved in promoting HCC development and malignant and cancer stemness characteristics [134,135,137,138,139]. When IGF/IGF-1R signaling was deactivated by miR-486-5p or siRNA-IGF-1R, the levels of STAT3, mTOR, and cMyc were reduced, and colony formation was suppressed [140]. IGF-1 treatment increased epithelial-mesenchymal transition (EMT) markers of migration and metastatic properties through STAT5 in HepG2 and Hep3B cells [141].

SOX2 is a transcription factor that regulates cancer stemness in a variety of cancers (liver cancer [142,143], colorectal cancer [144], breast cancer [145], gastrointestinal cancer [146], and pancreatic cancer [147]). Bu et al. have illustrated that in a subpopulation of HCC cells with cancer stemness properties and high levels of SOX2, IGF-1 production was upregulated [148]. Additionally, when IGF-1 function was blocked by miR-28-5p, the expressions of cancer stemness-related genes SOX2, OCT4, and NANOG were repressed [149]. Furthermore, high levels of SOX2 expression were found to be due to elevated levels of the IGF/IGF-1R signaling downstream proteins Akt and mTOR, which subsequently elevated HCC cancer stemness characteristics [143,150].

YAP is a key component in the Hippo signaling pathway, which responds to organ growth and development [151,152]. YAP acts as a co-transcription factor. In complexes with TAZ, YAP/TAZ bind to transcription factors such as TEADs (TEA domain family members) [153,154,155,156,157], RUNXs (runt-related transcription factor) [158,159], and TBX5 (T-box transcription factor 5, TBX5) [160] and induce gene transcription activity. The crosstalk between IGF/IGF-1R signaling and Hippo signaling was first demonstrated in HCC by Strassburger et al. in 2012. Samples from HCC patients indicated that the expression of YAP positively correlated with Akt phosphorylation, a downstream result of IGF/IGF-1R signaling [161]. Inhibition/induction of IGFs activation in both of mammalian and drosophila cell lines resulted in increasing/decreasing the level of the YAP active form, cell proliferation activity, and organ size [161,162]. IGF-1 or insulin treatment activated IGF/IGF-1R signaling then increased the YAP active form in HCC cell lines [163]. The role of YAP in promoting cancer stemness in liver cancer was demonstrated in several studies [164,165,166,167,168]. Nevertheless, direct evidence for IGF/IGF-1R signaling regulating liver cancer stemness through YAP activity has not yet been reported.

#### 3.2.2. IGF/IGF-1R Signaling Induces Expressions of Stemness-Related Cell Surface Markers

Cell markers that identify liver cancer stem cell populations include prominin-1 (CD133^+^), CD44^+^, CD13^+^, epithelial cell adhesion molecule (EpCAM^+^), CD90^+^, oval cell marker antibody 6 (OV6^+^), and aldehyde dehydrogenase (ALDH^+^) [169,170,171]. Liver cancer cells that express at least two markers are considered to be liver cancer stem cells (liver-CSCs).

It has been shown that upregulation of IGF/IGF-1R signaling results in an increase of cancer stemness properties in HCC. Autocrine IGF-1 signaling is responsible for regulating cancer stem cell (CSC) related markers (e.g., CD44^+^, ALDH^+^, and EpCAM^+^) in MHCC97H cells. When IGF-1 receptor was blocked by an IGF-1R specific inhibitor, the CSC-related markers in these cells were suppressed as well [148]. In addition, knockdown of IGF-1 by IGF-1-siRNA caused a decrease in the proportion of EpCAM^+^ cells and self-renewal ability [149]. On the other hand, high expression levels of IGF-2 were observed in both NANOG^+^-liver cancer stem cells, which were isolated from patient-derived primary HCC cells and in HCC cell lines [133]. Benabou et al. showed that overexpression of the Huh7-insulin receptor isoform A (IRA) caused an increase in the expression levels of IGF-2 and stemness markers CK19, CD133, CD44, EpCAM, and PROM1. Knocking down IGF-2 expression by siRNA repressed phosphorylation of IR [172]. Downstream of growth signaling (IGF-1R, EGFR), long noncoding RNA (lncRNA) H19 regulates cancer stemness and EMT markers in HepG2 and Hep3B2.1-7 cell lines. Overexpression/suppression of lncRNA H19 induced/reduced, respectively, the expression of stemness markers including Lin28 and Notch1 [173]. Pre-treatment with IGF-2 for 48 h upregulated mRNA and protein expression of CD133 and Bmi-1 as well as sphere formation in Huh7 cells [174]. In addition, studies have demonstrated that the expression of IGF-1R positively correlates with expression of both OCT4 and NANOG [133,134,138] and CD133 [132] in human HCC tissues and HCC cell lines. These findings suggest that liver-CSCs produce IGF, which then maintains stemness properties and CSC populations in liver cancer.

#### 3.2.3. IGF/IGF-1R Signaling Supports the Stem Cell Niche in HCC

The cancer stem cell niche is a special microenvironment where cancer stem cells reside and are maintained. It is made up of a variety of cell types, including cancer cells, cancer stem cells, immune cells, tumor-associated macrophages, mesenchymal stem cells, cancer-associated fibroblasts, and stroma cells [175,176,177]; thus, the regulatory signals in the niche are a combination of secreted factors from these cells including cytokines, chemokines, growth factors, hormones, microvesicles, and MMPs [177,178,179]. A direct role for IGF/IGF-1R signaling in regulating the stem cell niche [180,181] and cancer stem cell niche [126,182,183] has been reported.

#### 3.2.4. HBV Replication Promotes IGF/IGF-1R Signaling-Mediated Cancer Stemness Properties in HCC

**Figure 2:**
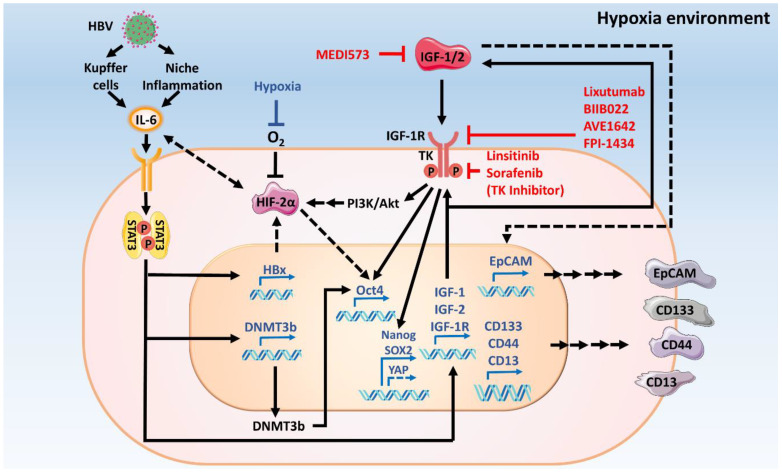
IGF/IGF-1R signaling induces cancer-related stemness in HCC. The formation of a hypoxic environment or the HBx protein can trigger and form a loop of IGF/IGF-1R signaling activation during HCC progression, leading to the activation of cancer stemness-related transcription factors and specific niches. Stimulation of these transcriptional pathways, such as Oct4, Nanog, and SOX2, and interactions in the niches increase cancer stemness and lead to the upregulation of stemness-associated cell surface markers, including EpCAM, CD133, CD44, and CD13. IGF-1, IGF-2, and IGF-1R targeting drugs and tyrosine kinase inhibitor (TKI) inhibit IGF/IGF-1R signaling, and thus discontinue the loop.

It is well known that HBV infection is a major initiating factor for HCC [184,185,186] and acts via activation of cell proliferation-related signaling pathways [186]. Upregulated expression of HBx—a regulatory protein for viral life cycle, has been linked to elevated levels of IGF-2 in patients with HBV-cirrhotic or noncirrhotic chronic liver disease [187] and in cell lines [69,120,188]. Studies on HBV-HCC sections found upregulation of IGF-2 mRNA in 50% of sections from HCC patients with HBV [187]. IGF-2 expression is regulated by HBx through the IGF-2 promotors p3 and p4 [69,120]. In addition, Nielson et al. reported that HBV suppresses the secretion of IGFBP1, activating IGF/IGF-1R signaling in HepG2 cells [189]. It was recently found that the replication of HBV is positively correlated to the expression of cancer stemness markers (EpCAM, CD44, CD133, NANOG, OCT4, and Sox2) [185,190,191], tumorigenesis, and self-renewal [191]. It was also demonstrated that patients with HBV-HCC have higher recurrence rates and higher expression levels of IGF-1R and cancer stemness markers. The activation of IGF/IGF-1R signaling by IGF-1 treatment leads to increased expression of NANOG and OCT4 in HBV positive-HCC cell lines, but not in HBV negative-HCC cell lines [134]. Taken together, these findings suggest that HBV induces cancer stemness properties through the activation of IGF/IGF-1R signaling (Figure 2).

#### 3.2.5. IGF/IGF-1R Signaling Induces Cancer Stemness Properties through Crosstalk with Cytokine Signaling

It has been reported that inflammatory cytokines work together with IGF/IGF-1R signaling in regulating cancer stem cell populations [192,193]. IL-6 is a cytokine involved in inflammatory mechanisms. Activation of IL-6/STAT3 signaling induces HCC cancer stemness [194] and promotes HCC cancer stem cell expansion [194]. The serum level of IL-6 is elevated in liver cancer patients who express high levels of NANOG and OCT4 [134,139,194]. IL-6 treatment induced the expression of OCT4, NANOG, SOX2 CD44, and HIF2α in both in vitro and in vivo models [139,194]. In addition, IL-6 treatment induced IGF-1 secretion and IGF-1R expression in NANOG/OCT4-HCC cell lines in a dose-dependent manner [134], suggesting crosstalk between IL-6/STAT3 signaling and IGF/IGF-1R signaling in HCC cancer stem cells (Figure 2).

The initiation of cancer stemness characteristics by inflammatory cytokines was demonstrated in HCC recently [138,195]. In an attempt to understand the role inflammation plays in stimulating the expression of stemness markers in HCC, Chang el al treated HepG2.2.15 and Hep3B cell lines with inflammation-conditioned medium (LPS-stimulated U937 condition medium) for 7 days and found increased levels of NANOG/OCT4/SOX2 mRNA and increased expression of NANOG/OCT4 in these cells. Moreover, inflamed-conditioned medium treatment not only increased IGF-1 and IGF-1R mRNA and protein levels but also increased IGF/IGF-1R signaling activity [138]. Results suggest a role for inflammation in promoting cancer stemness in HCC through IGF/IGF-1R signaling.

Daintain/Allograft Inflammatory factor-1 (AIF-1) is a macrophage-derived inflammatory cytokine. Daintain/AIF-1 is known to promote cell proliferation and migration in breast cancer [196,197] and to induce differentiation of hematopoietic stem cells [198]. An association between daintain/AIF-1 and HCC has also been observed recently. The expression of daintain/AIF-1 was found to be higher in HCC tissue and HCC cell lines than in adjacent normal liver tissue and normal hepatocytes. Suppression of daintain/AIF-1 expression by siRNA reduces cell proliferation and migration in HCC cell lines [193,199]. It was also found that daintain/AIF-1upregulates the secretion of IGF-1/IGF-2 and downregulates secretion of IGFBP3 in HepG2 cells [199]. Whether daintain/AIF-1 regulates cancer stemness in HCC through IGF/IGF-1R signaling is still unknown. Together, these findings demonstrate the role cytokines play in promoting liver cancer stemness through IGF/IGF-1R signaling.

### 3.3. IGF/IGF-1R Signaling Induces the EMT in HCC

Activation of IGF/IGF-1R signaling can promote an epithelial-mesenchymal transition (EMT), which is positively related to stemness, by interacting with other pathways or factors. High expression of IGFBP2 was observed in several highly metastatic HCC cell lines, such as HCCML3 and MHCC97H. IGFBP2 can contribute to activation of nuclear factor kappa B (NF-κB) and zinc finger E-Box binding homeobox 1 (ZEB1) transcription, increasing the level of mesenchymal markers [200]. In addition, IGF-1 can activate survivin (as well as N-cadherin, Vimentin, Snail1, Snail2, and Twist1) via the MEK/ERK and PI3K/AKT pathways and thereby induce EMT in HCC [141,201]. These results suggest that IGF/IGF-1R signaling may contribute to an increase in mesenchymal characteristics and HCC metastasis.

Another study has demonstrated that IGF/IGF-1R signaling in HBV-HCC promotes EMT by reducing epithelial cell markers. IGF-2 upregulation was observed in liver tissue from HBx homozygous (+/+) mice and in HepG2 cells transfected with HBV x gene (HepG2-HBx cells). In HepG2-HBx, downregulation of E-cadherin was also observed, and treatment with HepG2-HBx-conditioned medium reduced the level of E-cadherin in HepG2-Mock cells. Together, the research suggests that HBx-induced IGF-2 is essential to trigger EMT through the loss of E-cadherin [70].

## 4. The Stemness of HCC Contributes to the Limitation of Targeted Therapies

### 4.1. Current Targeted Treatments for HCC

Sorafenib (Nexavar^®^, Bayer HealthCare Pharmaceuticals, Leverkusen, Germany; Onyx Pharmaceuticals, CA, USA) is the first oral multitargeted tyrosine kinase inhibitor FDA-approved for the treatment of advanced HCC. Sorafenib, approved in 2008, inhibits multiple growth tyrosine kinase receptors (PDGFRs, VEGFRs, c-KIT, and RET) and their downstream tyrosine kinase cascades (Ras, Raf, Mek, and Erk) in both tumor cells and endothelial cells [202,203,204]. The median OS of patients treated with sorafenib was significantly longer than that of patients treated with placebo in both the SHARP trial and the Asia-Pacific trial (10.7 months vs. 7.9 months (*p* < 0.001) and 6.5 months vs. 4.2 months (*p* = 0.014), respectively) [11,12]. The recommended dose of sorafenib is 400 mg taken twice daily; however, due to a high percentage of adverse events in the sorafenib treatment group vs. the control group (SHARP trial: 26% vs. 7% and Asia-Pacific trial: 82% vs. 39%), the dose of sorafenib may be reduced to 400 mg once daily if necessary [11,12,203]. The biggest challenge of sorafenib treatment is that some patients acquire resistance to sorafenib, which limits the efficacy of therapy.

Lenvatinib (Lenvima^®^, Eisai Inc., Tokyo, Japan), the second drug approved for unresectable HCC as first-line treatment, was approved in 2018 after the success of a phase III trial on 1492 patients from 20 countries. The median OS of patients treated with lenvatinib (13.6 months) was similar to that of patients treated with sorafenib (12.4 months) [13]. Lenvatinib inhibits tumor growth by blocking several targets, including VEGFRs, PDGFRs, FGFs, SCF, PDGFR, c-KIT, and RET [205,206]. Depending on body weight, patients received either 12 mg daily (>60 kg) or 8 mg daily (<60 kg) [13]. Similar to sorafenib, patients may become resistant to lenvatinib treatment [207].

Regorafenib (Stivarga^®^, Bayer, Leverkusen, Germany) was approved as a second-line treatment for advanced liver cancer in 2017 [208]. Regorafenib was previously referred to as fluoro-sorafenib because its structure is similar to sorafenib; the only difference between the two molecules is a fluorine atom in the center phenyl ring [209]. Regorafenib was shown to target more tyrosine kinases than sorafenib, hence, it is expected to have better efficacy in patients with HCC. In addition to targeting the same growth receptor tyrosine kinases as sorafenib, regorafenib also inhibits SHP-1 (a negative regulator of STAT3 signaling), B-RafV600E (a mutated isoform of B-Raf), TIE2 (angiopoietin 1 receptor), and p38-MARK [209,210,211]. In a phase III trial of patients who did not respond to sorafenib, treatment with regorafenib prolonged the median OS from 7.8 months in the placebo arm to 10.6 months in the regorafenib arm. In addition, the risk of death was reduced by 37% [208]. The combination of regorafenib and immunotherapy agents has been suggested as third-line treatment for patients with HCC [212].

Cabozantinib (Cabometyx^®^ Exelixis, CA, USA) was approved for patients with HCC progressing after treatment with sorafenib, as a second-line treatment by the European Commission and the FDA in 2018 and 2019, respectively. Cabozantinib is an inhibitor of several tyrosine kinases involved in angiogenesis (VEGFR and TIE2), tumorigenesis (MET, AXL, FLT3, RET, and KIT), and metastasis (MER, AXL, TYRO3, and ROS1) [213,214]. Results from the cabozantinib phase III clinical trial demonstrated that cabozantinib prolongs the median OS of patients with HCC from 8.0 months with placebo to 10.2 months with cabozantinib. The median progression-free survival was also greater in patients treated with cabozantinib compared with patients treated with placebo (5.2 months vs. 1.9 months) [215].

Ramucirumab (Cyramza^®^, Eli Lily and Company, MA, USA) is a human recombinant immunoglobulin G1 monoclonal antibody that specifically binds to the VEGF-binding domain of VEGFR-2. The first phase 3 REACH trial recruited patients with HCC who were previously treated with sorafenib. However, it was determined that ramucirumab was only active in patients with high alpha-fetoprotein (AFP) levels. Therefore, the second trial (REACH-2) recruited 292 patients with HCC from 20 countries who were previously treated with sorafenib and had AFP ≥ 400 ng/mL [216]. In REACH-2, the median OS of patients in the ramucirumab group was significantly prolonged compared with the placebo group (8.5 months vs. 7.3 months). The progression-free survival was 1.6 months in the placebo group vs. 2.8 months in the ramucirumab group [217]. A meta-analysis of individual patient data from the REACH and REACH-2 clinical trials evaluated the median OS and progression-free survival of a pool of 291 Asian patients and a pool of 251 non-Asian patients. In both pools, the median OS of patients treated with ramucirumab was significantly longer than that of patients treated with placebo (Asian patients: 8.08 months vs. 4.76 months; non-Asian patients: 7.98 months vs. 5.22 months) [218]. In May 2019, the FDA approved ramucirumab for HCC patients who were previously treated with sorafenib and have an AFP ≥ 400 ng/mL. Ramucirumab has also shown efficacy in patients after lenvatinib failure. In a study of 12 patients who had failed lenvatinib treatment and had AFP levels ≥ 400 ng/mL (10 patients in ramucirumab group and 2 patients in the placebo group), after 6 weeks, 8/10 patients had stable disease and 3 patients showed reduced levels of AFP [219].

Nivolumab (Opdivo^®^, Bristol-Meyer Squibb, NY, USA), a recombinant human immunoglobulin monoclonal antibody, functions as an inhibitor of programmed cell death protein 1 (PD1). Nivolumab was approved by the FDA in 2017 as a second-line treatment for HCC patients previously treated with sorafenib, based on results of the CheckMate-040 phase I/II clinical trial. Of 262 patients enrolled, 202 patients (77%) completed treatment [220]. A total of 743 patients with HCC (nivolumab: *n* = 371 vs. sorafenib: *n* = 372) were recruited in the phase III CheckMate 459 trial to assess the safety and efficacy of nivolumab compared with sorafenib as first-line treatment of HCC. The median OS of patients in the nivolumab group was 16.4 months vs. 14.7 months for those in the sorafenib group (*p* = 0.0752) [221].

Pembrolizumab (Keytruda^®^, Merck Sharp & Dohme Corp., NJ, USA) is an immunoglobulin monoclonal anti-PD1 antibody. In 2019, based on results of an open-label, phase II clinical trial (KEYNOTE-224 trial), pembrolizumab was FDA-approved for patients with HCC who have been previously treated with sorafenib. The median OS in the KEYNOTE-224 trial was 12.9 months [222]. In the phase III KEYNOTE-240 trial, which included 413 patients from 27 countries, the median OS in the pembrolizumab group was 13.9 months vs. 10.6 months in the placebo group [223]. Similar to nivolumab, pembrolizumab failed to show a significant improvement in median OS in comparison with sorafenib as first-line therapy for HCC.

### 4.2. Treatment Limitations and Acquired Drug Resistance in HCC

The efficacy of targeted therapy for advanced HCC is limited, and acquired drug resistance is common in advanced HCC patients. The first-line treatment for patients with early stage HCC, surgical resection, confers a 5-year survival rate of 70%, but more than 50% of patients have advanced stage HCC at diagnosis [224]. Sorafenib has been the standard approved targeted therapy for advanced HCC in the previous one decade [225]. HCC patients treated with sorafenib had a significantly longer OS (10.7 months vs. 7.9 months) and time to progression (TTP; 5.5 months vs. 2.8 months), compared with patients treated with placebo, in the SHARP trial [226]. Although many patients with advanced HCC have a positive response to sorafenib at the beginning of therapy, patients commonly develop acquired drug resistance, which leads to disease progression [12].

Two kinds of drug resistance have been observed in previous studies. Some HCC patients have primary sorafenib resistance, meaning they do not respond to sorafenib from the beginning doses. Primary resistance is due to genetic heterogeneity, although the exact mechanisms responsible remain unclear. Most drug resistance of HCC patients is acquired sorafenib resistance, which means that patients initially respond to sorafenib but become resistant after a couple of weeks of treatment. Sorafenib is a multiple-target tyrosine kinase inhibitor (TKI) and can inhibit Raf-1, B-Raf, and kinase activity in the Ras/Raf/MEK/ERK signaling pathways, to further suppress tumor cell proliferation. The mechanism of drug resistance development may be related to changes in epigenetics in drug targets, upregulated transport processes, regulated cell death, or the tumor microenvironment [227]. The patient response rate of sorafenib is poor (0.7–3.0%); however, it is the recommend standard treatment for advanced HCC [228]. Hence, it is clear that alternative therapeutic strategies are needed.

Sorafenib combination treatment with adjuvant therapy for certain types of HCC has been proposed. It has been suggested that non-muscle myosin heavy chain IIA (MYH9) contributes to HBV-HCC. X protein expressed by HBV has been found to interact with MYH9 and induce its expression, leading to increased stemness. A recent study reported that targeting MYH9 increased survival in a HCC mouse model and promoted sorafenib sensitivity in HBx-silenced HCC cells [229]. Similarly, Witt-Kehati et al. showed that hepatoma cells with replicating HBV are less sensitive to sorafenib than those without. In addition, they studied the relationship between HBV and sorafenib resistance by investigating the activation state of the Raf-Mek-Erk pathway, one of the major targets of sorafenib, in the presence of HBV and in response to sorafenib. While blocking pErk in the pathway did not significantly reduce sorafenib resistance, knocking down phosphorylated mitogen-activated protein kinase 14 (pMAPK14) was found to rescue the efficacy of sorafenib in HBV-expressing hepatoma cells [230].

Targeting the IGF/IGFR signaling pathway is another possible therapeutic strategy. A 2015 study from Taiwan [231] examined the utility of targeting IL-6-IGF/IGFR-mediated signaling in HBV-HCC patients. They found that IGF/IGFR signaling was significantly increased in HBV-HCC tumor samples compared with NBNC, HCV, and BC-HCC tumor samples. In vitro experiments also showed that after treatment with IGF-1, the HBV-HCC cell line expressed significantly higher NANOG and OCT4, while the non-HBV-HCC cell line had no obvious response. Moreover, in the mouse models with xenografts of an HBV-HCC cell line, treatment with IL-6 increased expression of IGF-1, OCT4, and NANOG in liver tissues. The results suggest that increasing IGF/IGF-1R signaling further induces the stemness of HCC, particularly in HBV-HCC [134].

## 5. IGF/IGF-1R Signaling Responses for Targeted-Drugs Resistance in HCC

### 5.1. IGF/IGF-1 Signaling in Sorafenib Resistance

Increased IGF/IGF-1R signaling promotes sorafenib resistance through increasing the cancer stemness of cancer cells, upregulating the microRNAs, which regulate IGF/IGF1R signaling, or inducing in IGFBPs production.

Sorafenib (Nexavar), the first oral multiple targeting tyrosine kinase approved for the treatment of advanced liver cancer treatment in 2008, unfortunately has a low rate of disease control (43% and 35.3% in the SHARP and Asian-Pacific trials, respectively) [11,12]. One of the main causes of sorafenib resistance in HCC is increased growth factor signaling, including IGF/IGF-1R signaling. Although sorafenib inhibits several tyrosine kinases, it does not suppress IGF-1R phosphorylation in HCC cells [232,233]. Sorafenib treatment of Hep3B, HepG2, or Huh7 cells showed no change in levels of IGF-1R phosphorylation. Meanwhile, knocking down IGF-1R by shRNA or preventing IGF-1R phosphorylation by ceritinib enhanced the efficacy of sorafenib, reducing cell proliferation rate and tumor development [232]. Suppression of IGF/IGF-1R signaling induced sorafenib sensitivity in HCC cells. Co-treatment with sorafenib and PPP, an IGF-1R inhibitor, reduced cell proliferation and motility of HLF and PLC/PRL/5 cells compared with PPP alone [234].

In a study of sorafenib resistance, Tovar et al. demonstrated that the tumor initiation ability of cells derived from sorafenib-resistant tumors was higher than that of sorafenib sensitive tumor cells and non-treated tumor cells. Microarray analysis of sorafenib-resistant cells indicated an enrichment of IGF and FGF signaling cascades. In addition, inhibition of IGF and/or FGF signaling reduced cell survival in HCC cell lines and delayed tumor growth [235]. Other groups have illustrated that the expression of cancer stemness genes (OCT4, NANOG) induced by elevation of IGF-1/IGF-1R, correlates with high tumor recurrence in HCC patients [134] and sorafenib resistance. In addition, it was found that OCT4 can be upregulated by stimulating STAT3/DNA methyltransferases (DNMTs), and inhibiting DNMTs can increase sensitivity to sorafenib in HCC cell lines and in animal models [139].

Triggering sorafenib resistance through the regulation of IGF-1R by miRNA was reported in several studies. miRNAs are non-coding RNAs that play critical roles in post-transcriptional regulation of gene expression. MiRNAs bind to the target mRNA via base-pairing and then silence the gene [236]. Dysregulated miRNA expression has been identified in a variety of solid cancers [237]. MiR-122 is the most common miRNA in liver, and downregulation of MiR-122 has been correlated with hepatocarcinogenesis [238]. The level of miR-122 was found to be negatively correlated with IGF-1R mRNA in both sorafenib sensitive and sorafenib resistant patients. The functional target of miR-122 is IGF-1R, which is highly expressed in sorafenib resistant cells. Moreover, the level of miR-122 is significantly reduced in HCC sorafenib-resistant cells, while overexpression of this miRNA induces HCC cell sensitization to sorafenib by reducing IGF-1R [239]. Another study showed that elevated expression of the enhancer of zeste-2-polycomb repressive complex-2 (EZH2) subunit causes declining levels of miR-101, miR-122, miR-125b, and miR-139 in sorafenib resistant HCC cell lines. Treatments with miR-122, miR-125b, and miR-139 reduced the expression of IGF-1R and re-sensitized sorafenib-resistant cells to sorafenib. [240]. Another miRNA, miR-378a-3p, that regulates sorafenib-resistant property in HCC by silencing IGF-1R, was recently identified. The low level of miR-378a-3p is demonstrated in sorafenib-resistant cells. While, overexpression of this miRNA reversed sorafenib sensitivity in HCC sorafenib-resistant cells [241]. Taken together, the results suggest that downregulation of miRNA leads to overexpression of the IGF-1R gene and triggers sorafenib resistance in HCC cells.

Insulin-like growth factor binding proteins (IGFBPs) are modulators of IGF/IGF-1R signaling. IGFBPs bind to IGF ligands with high affinity and prevent IGF-mediated IG1R activation [242,243]. Downregulation of IGFBPs induces cell growth, migration, and invasion and promotes HCC development [99,104,244,245,246]. An interesting study has demonstrated the tumor suppressing capacity of conditioned media derived from human fetal MSCs (CM-hfMSC) containing high levels of IGFBPs. This medium enhanced the antitumor efficacy of sorafenib on HCC cells. In addition, CM-hfMSC depressed the growth of patient-derived cancer stem cells [125]. These findings suggest that recombinant IGFBPs might be a potential therapy for the treatment of liver cancer.

Interestingly, HBV replication also activates IGF/IGF-1R signaling and induces sorafenib resistance. We previously reviewed the correlation between HBV replication and activation of IGF/IGF-1R signaling in HBV-related HCC. Because upregulation of HBx in HBV-replicating HCC cells promotes cancer stemness properties, which confer sorafenib resistance in liver cancer, the mechanism of sorafenib resistance in HBV-replicating cells was examined. Findings demonstrated that HBx-ΔC1 upregulation in HCC-HBV cell lines (Bel-7402 and SMMC-7721) suppressed the apoptosis cascade proteins (Bax and Bcl2) and cleaved proteins (cleaved caspase 3, cleaved caspase 9 and cleaved PARP), hence preventing cell death programs under sorafenib treatment. In parallel, upregulation of HBx-ΔC1 led to upregulation of IGF-2 production, which induced cell proliferation [191]. Furthermore, a decline in IGFBP1 production was found in HBV-replicating cells. Inhibition of HBx in HBV-HCC cells by doxycycline resulted in reduced IGFBP production and expression [189]. This finding suggests that when HBV integrates into the cell genome, it leads to dysregulation of IGF/IGF-1R signaling in liver cancer. In addition, the presence of HBV DNA in the HCC genome was found in cases of sorafenib resistance mediated via activation of other signaling pathways including MAPK14 [230], NF-κB [247], and Wnt [229], which crosstalk with IGF/IGF-1R signaling [235,248,249].

### 5.2. IGF/IGF-1 Signaling in Other Targeted-Drug Resistances

Similar to sorafenib, regorafenib is a small-molecule tyrosine kinase inhibitor, which was FDA-approved for advanced liver cancer treatment in 2017 as a second-line treatment. However, resistance to regorafenib via IGF/IGF-1R signaling was reported in 2015. Lippoliss et al. demonstrated that regorafenib inhibits cell growth, metastasis, and invasion by increasing expression of pro-apoptotic proteins (Bim, Bad) and reducing levels of activated growth signaling proteins (p-Erk, p-c-Myc, p-STAT3, and p-p38). IGF-1 treatment antagonized the inhibitory effects of regorafenib in HCC cell lines [250]. The understanding of regorafenib resistance in HCC has continued to evolve. Suemura et al. used a CRISPR/Cas9-based loss-of-function genetic screen to identify molecules involved in regorafenib resistance in HCC and found that the Hippo pathway is involved in regorafenib resistance in HCC cells. Blocking the Hippo signaling mediator YAP using a specific inhibitor (Verteporfin) reversed the insensitivity of HCC regorafenib-resistant cells to become regorafenib-sensitive [251]. Meanwhile, the Hippo and IGF/IGF-1R signaling pathways were demonstrated to be linked in conferring sorafenib resistance in HCC cells [163]. Additional investigations into mechanisms behind regorafenib resistance in HCC found that resistance is mediated via proteins controlling the cell cycle and proliferation—NF-κB, STAT3, Akt, cMyc [252,253,254,255]. Thus, the IGF/IGF-1R signaling may be a potential target for combination treatment with regorafenib.

Gefitinib (Iressa^®^, AstraZeneca Pharmaceuticals, Cambridge, England) is a tyrosine kinase inhibitor that targets epidermal growth factor receptor (EGFR). Gefitinib treatment of HCC Mahlavu cells raised the phosphorylation level of IGF-1R and downstream p-Akt. It was also demonstrated that nuclear translocation of IGF-1R triggered gefitinib resistance in these cells [132]. Moreover, inhibiting IGF-1R caused HCC cells to become sensitive to gefitinib. Mouthon et al. demonstrated that activation of both EGFR and IGF-1R by EGF and IGF, respectively, resulted in increased phosphorylation levels of Erk and Akt in Hep3B and HepG2 cell lines. Gefitinib co-treatment with EGF or IGF-2 suppressed phosphorylation of Erk, but not of Akt. Blocking IGF-1R with the selective inhibitor AG1024 sensitized HCC cells to gefitinib by increasing the level of cleaved PARP [256]. In addition, combination treatment with gefitinib and sorafenib controlled HCC cell growth better than sorafenib alone [257]. These findings suggest that suppressing IGF/IGF-1R signaling increases the efficacy of gefitinib in HCC cells.

Evidence for IGF/IGF-1R signaling conferring lenvatinib resistance in HCC still has not been investigated; however, some IGF/IGF-1R-crosstalked signaling have been found to be involved in lenvatinib resistance. The hepatocyte growth factor (HGF), which plays a central role in inducing cell proliferation, angiogenesis and tumorigenesis, has been determined to promote lenvatinib resistance through the downstream targets Akt and Erk. Inactivation of this signal can reverse the sensitivity of HCC cells to lenvatinib [258].

## 6. IGF/IGF-1 Signaling as a Potential Target for the Treatment of Advanced HCC

The concept of targeting IGF/IGF-1 signaling has been studied in multiple clinical trials, several of which failed. Reasons these clinical trials failed include issues with patient recruitment, patient time investment, drug toxicity, and lack of efficacy. In Table 1, we review past clinical trials that targeted IGF/IGF-1 signaling. Moreover, we provide a novel perspective on the reasons for failure.

### 6.1. Former Trials Targeting IGF/IGF-1R Signaling in HCC

The efficacy and safety of cixutumumab (IMC-A12), a monoclonal IGF-1R targeting antibody [263], were evaluated in a phase II clinical trial [259]. A total of 24 patients with advanced HCC, Child-Pugh A-B8, and KPS ≥ 60% were recruited into the trial and received 6 mg/kg of cixutumumab weekly [264]. Progression free survival (PFS), OS, and tumor size were analyzed. Unfortunately, the 4-month PFS was inferior to that of placebo, there was no evidence of tumor shrinkage, and no patients had an objective response. In addition, IGF-1R was not significantly correlated with the outcome of patients. Cixutumumab alone did not show enough clinically meaningful activity in these unselected patients with HCC to continue the trial.

A phase I clinical trial assessed the combination of cixutumumab and sorafenib in HCC patients. The investigators recruited 21 unselected patients with advanced HCC and Child-Pugh A to B7. Patients received sorafenib 400 mg twice a day and escalating doses of cixutumumab (2, 4, or 6 mg/kg weekly), and PFS and OS were recorded [260]. Although the toxicity profile was similar to that of sorafenib monotherapy, the clinical efficacy of the combination of cixutumumab and sorafenib remained limited.

BIIB022 is an anti-IGF-1R monoclonal antibody that blocks the binding of both IGF-1 and IGF-2 to IGF-1R. Since it does not contain Fc effector function, it may minimize toxicities in healthy tissues expressing IGF-1R [261]. A phase Ib trial was conducted to evaluate the safety and tolerability of BIIB022 in combination with sorafenib in advanced HCC patients. However, results have not yet been published.

### 6.2. Reasons for Failing Trials and Potential Improvement of IGF/IGF-1R Targeted Therapies in HBV-HCC

To optimize the trial design in the future, we strongly propose that two factors should be considered.

The etiology of HCC should be considered in the inclusive criteria of clinical trials. Lack of etiological stratification in patient recruitment may be a reason for the failure of all of the clinical trials mentioned above. IGF/IGF-1R signaling is now known to be significantly increased in HBV-HCC, especially, compared with other types of HCC [134,139]. Therefore, while the therapies targeting IGF/IGF-1R signaling showed limited clinical activity on HCC with unspecific etiology, we strongly suggested that they would be efficacious in HBV-HCC. We believe that the etiology of HCC should be considered an important factor when recruiting patients for enrollment in clinical trials. Specifically, selecting patients with HBV-HCC for the clinical trials assessing IGF-1R-targeting monoclonal antibodies and sorafenib combination therapies may make these trials more likely to be successful.

In addition, administrating the IGF/IGF-1R signaling-targeted drugs via the hepatic artery, the artery that carries blood directly to the liver tumors, can provide a liver-specific therapeutic strategy for HCC patients. Transarterial chemoembolization (TACE) is a kind of transarterial infusion method and is widely used in liver cancer patients who cannot undergo resection surgery or have tumor recurrence. During the process, chemical agents are infused into the artery that feed tumors. After infusion, the embolization in the following step block the artery and improve the retention of the drugs in the artery that supply tumors [265]. IGF/IGF-1R signaling manipulates cell proliferation, organ development, and regeneration throughout the body, making it hard to become a therapeutic target without hurting its physiological function, and since IGF/IGF-1R signaling occurs not only in the liver but also in other organs, delivering drugs targeting IGF/IGF-1R signaling via oral or intravenous routes might cause harmful effects to the other organs [266]. We highly recommend that transarterial infusion, such as TACE, would be the best way to minimize the risks of side effects triggered by drugs that target IGF/IGF-1R signaling and improve the efficacy of the drugs.

## 7. Conclusions

In this review, we highlight the evidence for dysregulated IGF/IGF-1R signaling in HCC. In the pathophysiology of HCC, numerous factors can initiate carcinogenesis, including hepatitis virus infection, microenvironmental factors, and imbalance of IGF-1R modulators. As tumors grow, a portion of cancer cells acquire stemness properties due to induction of critical pluripotent transcription factors by IGF/IGF-1R signaling, followed by functional alterations in these cells. Cell stemness may make cells resistant to targeted therapies, including first-line sorafinib and lenvatinib treatments in HCC. Recently, IL-6–DNMT3b–mediated OCT4 expression was found to correlate with tumor early recurrence and poor prognosis of HCC patients [139]. Activation of IGF/IGF-1R signaling induces the stemness properties of HBV-HCC [134]. Based on these insights, diminishing cancer stemness can be further explored through targeting both IGF/IGF-1R signaling molecules and epigenetic modulators. Although some trials of IGF-1R targeting agents have shown safety issues or limited efficacy (Table 1), it is hoped that further investigation into this strategy of combining anti-cancer drugs with agents that inhibit IGF/IGF-1R signaling will yield effective treatment for HBV-associated HCC.

## Figures and Tables

**Table 1:** Agents that target IGFs/IGF-1R in development for liver cancer treatments.

Compound	Locations of Clinical Trial	Mechanism of Action	Phase	Combination Treatment	No. of Participants (*n*)	HCC Etiology of Participants	Conclusion	Clinical Trial No. or Ref.	Year of Clinical Trial
**Monoclonal antibodies**									
MEDI-573	United States	Anti IGFI and IGFII	Phase 1b	Combination with sorafenib	6	Unspecific	Safety population included all participants who received any amount of study treatment.	NCT01498952	2012–2013
Cixutumumab (IMC-A12)	United States	Anti IGF-1R	Phase II	Single	24	Unspecific	Cixutumumab alone did not show the clinical meaningful activity in recruited patients with HCC.	NCT00639509 [259]	2008–2011
Cixutumumab (IMC-A12)	United States	Anti IGF-1R	Phase I	Combination with sorafenib	21	Unspecific	Combination of cixutumumab and sorafenib showed limited clinical efficacy in patients with HCC.	NCT01008566 [260]	2009–2016
BIIB022	United States Singapore Taiwan United Kingdom	Anti IGF-1R	Phase Ib	Combination with sorafenib	40	Unspecific	The dose of BIIB022 was established for clinical trial phase II.	NCT00956436 [261]	2009–2011
AVE1642	France	Anti IGF-1R	Phase I	Single and Combination with sorafenib	13	Unspecific	AVE1642 can be safely combined with active doses of sorafenib.	NCT00791544 [262]	2008–2009
FPI-1434	United States Australia Canada	Anti IGF-1R	Phase I	Single	38	Unspecific	Recruiting.	NCT03746431	2019–2022
**Inhibitors**									
Linsitinib (OSI-906)	United States Belgium France Germany Hong Kong Italy Korea Singapore Spain Taiwan	IGF-1R phosphorylation inhibitor	Phase II	Single	23	Unspecific	Terminated due to safety issue observed.	NCT01101906	2011

## Data Availability

Not applicable.

## Abbreviations

AFR	Albumin-to-Fibrinogen Ratio
AIF-1	Allograft Inflammatory Factor-1
Akt	Ak Strain Transforming
ALDH	Aldehyde Dehydrogenase
Bax	BCL2 Associated X
BC-HCC	Hepatitis B and C-Hepatocellular Carcinoma
Bcl2	BCL2 Apoptosis Regulator
CAF	Cancer-Associated Fibroblasts
c-KIT	*KIT* Proto-Oncogene
CM	Conditioned Media
CRISPR/Cas9	Clustered Regularly Interspaced Short Palindromic Repeats Cas9
CSC	Cancer Stem Cell
DAAs	Direct-Acting Antivirus
DENA	Diethylnitrosamine
DNA	Deoxyribonucleic Acid
DNMT	DNA Methyltransferase
EMT	Epithelial-Mesenchymal Transition
EZH2	Enhancer of Zeste 2 Polycomb Repressive Complex 2 Subunit
FDA	U.S. Food and Drug Administration
FGF	Fibroblast Growth Factor
FLT3	FMS-Like Tyrosine Kinase 3
GH	Growth Hormone
HBeAg	Hepatitis B E-Antigen
HBV	Hepatitis B Virus
HBx	Hepatitis B Virus X Protein
HCC	Hepatocellular Carcinoma
HCV	Hepatitis C Virus
hfMSC	Human Fetal Mesenchymal Stem Cell
HIF	Hypoxia-Inducible Factor
HIV	Human Immunodeficiency Virus
IGF-1/2	Insulin-Like Growth Factor 1/2
IGF-1R	Insulin-Like Growth Factor 1 Receptor
IGFBP	Insulin-Like Growth Factor Binding Protein
IL-6	Interleukin 6
INSR-A	*Insulin Receptor*-A
Jak	Janus Kinase
KPS	Karnofsky Performance Status Scale
lncRNA	Long Non-Coding RNA
MAPK (Erk)	Mitogen-Activated Protein Kinase
MAPKK (Mek)	Mitogen-Activated Protein Kinase Kinase
MER	*Mer* Receptor Tyrosine Kinase
MET	Receptor Tyrosine Kinase
miRNA	MicroRNA
MMP	Matrix Metalloproteinases
mRNA	Messenger RNA
MSC	Mesenchymal Stem Cell
mTOR	Mechanistic Target of Rapamycin
NANOG	*Nanog* Homeobox
NBNC	Non-B Non-C Hepatocellular Carcinoma
NFκB	Nuclear Factor Kappa Beta
NGF	Nerve Growth Factor
OCT4	Octamer-Binding Transcription Factor 4
ORF	Open Reading Frame
OS	Overall Survival
OV6	Oval Cell Marker Antibody 6
PD1	Programmed Cell Death Protein 1
PDGFR	Platelet Derived Growth Factor Receptor
PFS	Progression Free Survival
PI-3K	Phosphatidylinositol-3-Kinase
PROM1 (CD133)	Prominin-1
PSA	Prostate-Specific Antigen
Raf	Rapidly Accelerated Fibrosarcoma
Rb pathway	Retinoblastoma Pathway
REACH	Research to Assess Svs [Spiration^®^ Valve System]
RET	Rearranged During Transfection
rhGH	Recombinant Human GH
ROS1	Ros Proto-Oncogene 1
RUNX	Runt-Related Transcription Factor
SCF	Stem Cell Factor
SHARP	Study of Heart and Renal Protection
SiRNA	Silent RNA
SOX2	Sex Determining Region Y-Box2
STATs	Signal Transducer and Activator of Transcription
TAZ	Tafazzin
TBX5	T-Box Transcription Factor 5
TCGA	The Cancer Genome Atlas
TEAD	Tea Domain Family Member
TGF-β	Transforming Growth Factor Beta-1
TIA-1	T-Cell-Restricted Intracellular Antigen-1
TIE2	Tek Receptor Tyrosine Kinase
TYRO3	Tyrosine-Protein Kinase Receptor 3
VEGFR	Vascular Endothelial Growth Factor Receptor
WHO	World Health Organization
Wnt	Wingless-Related Integration Site
YAP	Yes-Associated Protein
ZEB1	Zinc Finger E-Box Binding Homeobox 1

## References

[1] European Association for the Study of the Liver (2018). EASL clinical practice guidelines: Management of hepatocellular carcinoma. J. Hepatol..

[2] Bray F., Ferlay J., Soerjomataram I., Siegel R.L., Torre L.A., Jemal A. (2018). Global cancer statistics 2018: Globocan estimates of incidence and mortality worldwide for 36 cancers in 185 countries. CA Cancer J. Clin..

[3] Fujiwara N., Friedman S.L., Goossens N., Hoshida Y. (2018). Risk factors and prevention of hepatocellular carcinoma in the era of precision medicine. J. Hepatol..

[4] Villanueva A. (2019). Hepatocellular carcinoma. N. Engl. J. Med..

[5] Mak L.-Y., Cruz-Ramón V., Chinchilla-López P., Torres H.A., LoConte N.K., Rice J.P., Foxhall L.E., Sturgis E.M., Merrill J.K., Bailey H.H. (2018). Global epidemiology, prevention, and management of hepatocellular carcinoma. Am. Soc. Clin. Oncol. Educ. Book.

[6] Chang I.C., Huang S.F., Chen P.J., Chen C.L., Chen C.L., Wu C.C., Tsai C.C., Lee P.H., Chen M.F., Lee C.M. (2016). The hepatitis viral status in patients with hepatocellular carcinoma: A study of 3843 patients from taiwan liver cancer network. Medicine.

[7] Stanaway J.D., Flaxman A.D., Naghavi M., Fitzmaurice C., Vos T., Abubakar I., Abu-Raddad L.J., Assadi R., Bhala N., Cowie B. (2016). The global burden of viral hepatitis from 1990 to 2013: Findings from the global burden of disease study 2013. Lancet.

[8] World-Health-Organization (2017). Global Hepatitis Report, 2017.

[9] World-Health-Organization (2016). Global Health Sector Strategy on Viral Hepatitis 2016–2021: Towards Ending Viral Hepatitis.

[10] Yang J.D., Hainaut P., Gores G.J., Amadou A., Plymoth A., Roberts L.R. (2019). A global view of hepatocellular carcinoma: Trends, risk, prevention and management. Nat. Rev. Gastroenterol. Hepatol..

[11] Llovet J.M., Ricci S., Mazzaferro V., Hilgard P., Gane E., Blanc J.F., de Oliveira A.C., Santoro A., Raoul J.L., Forner A. (2008). Sorafenib in advanced hepatocellular carcinoma. N. Engl. J. Med..

[12] Cheng A.L., Kang Y.K., Chen Z., Tsao C.J., Qin S., Kim J.S., Luo R., Feng J., Ye S., Yang T.S. (2009). Efficacy and safety of sorafenib in patients in the asia-pacific region with advanced hepatocellular carcinoma: A phase iii randomised, double-blind, placebo-controlled trial. Lancet Oncol..

[13] Kudo M., Finn R.S., Qin S., Han K.H., Ikeda K., Piscaglia F., Baron A., Park J.W., Han G., Jassem J. (2018). Lenvatinib versus sorafenib in first-line treatment of patients with unresectable hepatocellular carcinoma: A randomised phase 3 non-inferiority trial. Lancet.

[14] Mulvihill M.J., Cooke A., Rosenfeld-Franklin M., Buck E., Foreman K., Landfair D., O’Connor M., Pirritt C., Sun Y., Yao Y. (2009). Discovery of osi-906: A selective and orally efficacious dual inhibitor of the igf-1 receptor and insulin receptor. Future Med. Chem..

[15] Hua H., Kong Q., Yin J., Zhang J., Jiang Y. (2020). Insulin-like growth factor receptor signaling in tumorigenesis and drug resistance: A challenge for cancer therapy. J. Hematol. Oncol..

[16] Valaydon Z.S., Locarnini S.A. (2017). The virological aspects of hepatitis b. Best Pract. Res. Clin. Gastroenterol..

[17] Colgrove R., Simon G., Ganem D. (1989). Transcriptional activation of homologous and heterologous genes by the hepatitis b virus x gene product in cells permissive for viral replication. J. Virol..

[18] Zhang X., Zhang H., Ye L. (2006). Effects of hepatitis b virus x protein on the development of liver cancer. J. Lab. Clin. Med..

[19] Weil R., Sirma H., Giannini C., Kremsdorf D., Bessia C., Dargemont C., Bréchot C., Israël A. (1999). Direct association and nuclear import of the hepatitis b virus x protein with the nf-κb inhibitor iκbα. Mol. Cell. Biol..

[20] Forgues M., Marrogi A.J., Spillare E.A., Wu C.G., Yang Q., Yoshida M., Wang X.W. (2001). Interaction of the hepatitis b virus x protein with the crm1-dependent nuclear export pathway. J. Biol. Chem..

[21] Kidd-Ljunggren K., Oberg M., Kidd A.H. (1997). Hepatitis b virus x gene 1751 to 1764 mutations: Implications for hbeag status and disease. J. Gen. Virol..

[22] Feitelson M.A., Duan L.-X., Guo J., Sun B., Woo J., Steensma K., Horiike N., Blumberg B.S. (1995). X region deletion variants of hepatitis b virus in surface antigen-negative infections and non-a, non-b hepatitis. J. Infect. Dis..

[23] Gearhart T.L., Bouchard M.J. (2010). The hepatitis b virus x protein modulates hepatocyte proliferation pathways to stimulate viral replication. J. Virol..

[24] Ayub A., Ashfaq U.A., Haque A. (2013). Hbv induced hcc: Major risk factors from genetic to molecular level. Biomed. Res. Int..

[25] Tsai K.-N., Kuo C.-F., Ou J.-H.J. (2018). Mechanisms of hepatitis b virus persistence. Trends Microbiol..

[26] Umar M., Hamama Tul B., Umar S., Khan H.A. (2013). Hbv perinatal transmission. Int. J. Hepatol..

[27] McMahon B.J., Alward W.L., Hall D.B., Heyward W.L., Bender T.R., Francis D.P., Maynard J.E. (1985). Acute hepatitis b virus infection: Relation of age to the clinical expression of disease and subsequent development of the carrier state. J. Infect. Dis..

[28] Tassopoulos N.C., Papaevangelou G.J., Sjogren M.H., Roumeliotou-Karayannis A., Gerin J.L., Purcell R.H. (1987). Natural history of acute hepatitis b surface antigen-positive hepatitis in greek adults. Gastroenterology.

[29] Blach S., Zeuzem S., Manns M., Altraif I., Duberg A.-S., Muljono D.H., Waked I., Alavian S.M., Lee M.-H., Negro F. (2017). Global prevalence and genotype distribution of hepatitis c virus infection in 2015: A modelling study. Lancet Gastroenterol. Hepatol..

[30] Hajarizadeh B., Grebely J., Dore G.J. (2013). Epidemiology and natural history of hcv infection. Nat. Rev. Gastroenterol. Hepatol..

[31] Heffernan A., Cooke G.S., Nayagam S., Thursz M., Hallett T.B. (2019). Scaling up prevention and treatment towards the elimination of hepatitis c: A global mathematical model. Lancet.

[32] Wang M., Kaufman R.J. (2016). Protein misfolding in the endoplasmic reticulum as a conduit to human disease. Nature.

[33] Vescovo T., Refolo G., Vitagliano G., Fimia G.M., Piacentini M. (2016). Molecular mechanisms of hepatitis c virus–induced hepatocellular carcinoma. Clin. Microbiol. Infect..

[34] Dash S., Aydin Y., Widmer K.E., Nayak L. (2020). Hepatocellular carcinoma mechanisms associated with chronic hcv infection and the impact of direct-acting antiviral treatment. J. Hepatocell. Carcinoma.

[35] Liu C.J., Chu Y.T., Shau W.Y., Kuo R.N., Chen P.J., Lai M.S. (2014). Treatment of patients with dual hepatitis c and b by peginterferon α and ribavirin reduced risk of hepatocellular carcinoma and mortality. Gut.

[36] Marot A., Belaid A., Orlent H., Sersté T., Michielsen P., Colle I., Laleman W., de Galocsy C., Reynaert H., D’Heygere F. (2017). Characteristics of patients with hepatitis b virus and hepatitis c virus dual infection in a western european country: Comparison with monoinfected patients. Clin. Res. Hepatol. Gastroenterol..

[37] Fong T.L., Di Bisceglie A.M., Waggoner J.G., Waggoner J.G., Banks S.M., Hoofnagle J.H. (1991). The significance of antibody to hepatitis c virus in patients with chronic hepatitis b. Hepatology.

[38] Al Karawi M.A., Mesa G.A. (1997). Dual infection with hepatitis c and b viruses: Clinical and histological study in saudi patients. Hepatogastroenterology.

[39] Thio C.L., Seaberg E.C., Skolasky R., Phair J., Visscher B., Muñoz A., Thomas D.L. (2002). Hiv-1, hepatitis b virus, and risk of liver-related mortality in the multicenter cohort study (macs). Lancet.

[40] Soriano V., Vispo E., Labarga P., Medrano J., Barreiro P. (2010). Viral hepatitis and hiv co-infection. Antiviral Res..

[41] Maier I., Wu G.Y. (2002). Hepatitis c and hiv co-infection: A review. World J. Gastroenterol..

[42] Smith K.B., Smith M.S. (2016). Obesity statistics. Prim. Care.

[43] Rui R., Lou J., Zou L., Zhong R., Wang J., Xia D., Wang Q., Li H., Wu J., Lu X. (2012). Excess body mass index and risk of liver cancer: A nonlinear dose-response meta-analysis of prospective studies. PLoS ONE.

[44] Hashimoto M., Tashiro H., Kobayashi T., Kuroda S., Hamaoka M., Ohdan H. (2017). Influence of higher bmi for hepatitis b- and c-related hepatocellular carcinomas. Langenbecks Arch. Surg..

[45] Nugent C., Younossi Z.M. (2007). Evaluation and management of obesity-related nonalcoholic fatty liver disease. Nat. Clin. Pract. Gastroenterol. Hepatol..

[46] White D.L., Kanwal F., El-Serag H.B. (2012). Association between nonalcoholic fatty liver disease and risk for hepatocellular cancer, based on systematic review. Clin. Gastroenterol. Hepatol..

[47] Kanwal F., Kramer J.R., Mapakshi S., Natarajan Y., Chayanupatkul M., Richardson P.A., Li L., Desiderio R., Thrift A.P., Asch S.M. (2018). Risk of hepatocellular cancer in patients with non-alcoholic fatty liver disease. Gastroenterology.

[48] Alexander M., Loomis A.K., van der Lei J., Duarte-Salles T., Prieto-Alhambra D., Ansell D., Pasqua A., Lapi F., Rijnbeek P., Mosseveld M. (2019). Risks and clinical predictors of cirrhosis and hepatocellular carcinoma diagnoses in adults with diagnosed nafld: Real-world study of 18 million patients in four european cohorts. BMC Med..

[49] Kim G.-A., Lee H.C., Choe J., Kim M.-J., Lee M.J., Chang H.-S., Bae I.Y., Kim H.-K., An J., Shim J.H. (2018). Association between non-alcoholic fatty liver disease and cancer incidence rate. J. Hepatol..

[50] Pais R., Fartoux L., Goumard C., Scatton O., Wendum D., Rosmorduc O., Ratziu V. (2017). Temporal trends, clinical patterns and outcomes of nafld-related hcc in patients undergoing liver resection over a 20-year period. Aliment. Pharmacol. Ther..

[51] Younossi Z., Stepanova M., Ong J.P., Jacobson I.M., Bugianesi E., Duseja A., Eguchi Y., Wong V.W., Negro F., Yilmaz Y. (2019). Nonalcoholic steatohepatitis is the fastest growing cause of hepatocellular carcinoma in liver transplant candidates. Clin. Gastroenterol. Hepatol..

[52] Deng Z.-L., Ma Y. (1998). Aflatoxin sufferer and p53 gene mutation in hepatocellular carcinoma. World J. Gastroenterol..

[53] Macé K., Aguilar F., Wang J.S., Vautravers P., Gómez-Lechón M., Gonzalez F.J., Groopman J., Harris C.C., Pfeifer A.M. (1997). Aflatoxin b1-induced DNA adduct formation and p53 mutations in cyp450-expressing human liver cell lines. Carcinogenesis.

[54] Kew M.C. (2013). Synergistic interaction between aflatoxin and hepatitis b virus in hepatocarcinogenesis. Liver Int..

[55] Stern M.C., Umbach D.M., Yu M.C., London S.J., Zhang Z.-Q., Taylor J.A. (2001). Hepatitis b, aflatoxin b1, and p53 codon 249 mutation in hepatocellular carcinomas from guangxi, people’s republic of china, and a meta-analysis of existing studies. Cancer Epidemiol. Biomark. Prev..

[56] Chittmittrapap S., Chieochansin T., Chaiteerakij R., Treeprasertsuk S., Klaikaew N., Tangkijvanich P., Komolmit P., Poovorawan Y. (2013). Prevalence of aflatoxin induced p53 mutation at codon 249 (r249s) in hepatocellular carcinoma patients with and without hepatitis b surface antigen (hbsag). Asian Pac. J. Cancer Prev..

[57] Niemelä O., Parkkila S., Pasanen M., Iimuro Y., Bradford B., Thurman R.G. (1998). Early alcoholic liver injury: Formation of protein adducts with acetaldehyde and lipid peroxidation products, and expression of cyp2e1 and cyp3a. Alcohol Clin. Exp. Res..

[58] Tsukamoto H., Horne W., Kamimura S., Niemelä O., Parkkila S., Ylä-Herttuala S., Brittenham G.M. (1995). Experimental liver cirrhosis induced by alcohol and iron. J. Clin. Investig..

[59] Ganne-Carrie N., Nahon P. (2019). Hepatocellular carcinoma in the setting of alcohol-related liver disease. J. Hepatol..

[60] De Minicis S., Brenner D.A. (2008). Oxidative stress in alcoholic liver disease: Role of nadph oxidase complex. J. Gastroenterol. Hepatol..

[61] Zago A., Moreira F.P., Jansen K., Lhullier A.C., da Silva R.A., de Oliveira J.F., Medeiros J.R.C., Colpo G.B., Portela L.V., Lara D.R. (2016). Alcohol use disorder and inflammatory cytokines in a population sample of young adults. Drug Alcohol Depend..

[62] Neupane S.P., Skulberg A., Skulberg K.R., Aass H.C.D., Bramness J.G. (2016). Cytokine changes following acute ethanol intoxication in healthy men: A crossover study. Mediat. Inflamm..

[63] Crews F.T., Bechara R., Brown L.A., Guidot D.M., Mandrekar P., Oak S., Qin L., Szabo G., Wheeler M., Zou J. (2006). Cytokines and alcohol. Alcohol Clin. Exp. Res..

[64] An L., Wang X., Cederbaum A.I. (2012). Cytokines in alcoholic liver disease. Arch. Toxicol..

[65] Turati F., Galeone C., Rota M., Pelucchi C., Negri E., Bagnardi V., Corrao G., Boffetta P., La Vecchia C. (2014). Alcohol and liver cancer: A systematic review and meta-analysis of prospective studies. Ann. Oncol..

[66] Julien J., Ayer T., Bethea E.D., Tapper E.B., Chhatwal J. (2020). Projected prevalence and mortality associated with alcohol-related liver disease in the USA, 2019–2040: A modelling study. Lancet Public Health.

[67] De Minicis S., Agostinelli L., Rychlicki C., Sorice G.P., Saccomanno S., Candelaresi C., Giaccari A., Trozzi L., Pierantonelli I., Mingarelli E. (2014). Hcc development is associated to peripheral insulin resistance in a mouse model of nash. PLoS ONE.

[68] Adamek A., Kasprzak A. (2018). Insulin-like growth factor (igf) system in liver diseases. Int. J. Mol. Sci..

[69] Tang S., Hu W., Hu J., Wu S., Li J., Luo Y., Cao M., Zhou H., Jiang X. (2015). Hepatitis b virus x protein promotes p3 transcript expression of the insulin-like growth factor 2 gene via inducing hypomethylation of p3 promoter in hepatocellular carcinoma. Liver Int..

[70] Ha H.L., Kwon T., Bak I.S., Erikson R.L., Kim B.Y., Yu D.Y. (2016). Igf-ii induced by hepatitis b virus x protein regulates emt via sumo mediated loss of e- cadherin in mice. Oncotarget.

[71] Kadakia R., Josefson J. (2016). The relationship of insulin-like growth factor 2 to fetal growth and adiposity. Horm. Res. Paediatr..

[72] Oberbauer A.M. (2013). The regulation of igf-1 gene transcription and splicing during development and aging. Front. Oncol..

[73] Duguay S.J. (1999). Post-translational processing of insulin-like growth factors. Horm. Metab. Res..

[74] Abdel-Wahab R., Shehata S., Hassan M.M., Habra M.A., Eskandari G., Tinkey P.T., Mitchell J., Lee J.-S., Amin H.M., Kaseb A.O. (2015). Type i insulin-like growth factor as a liver reserve assessment tool in hepatocellular carcinoma. J. Hepatol..

[75] Weroha S.J., Haluska P. (2012). The insulin-like growth factor system in cancer. Endocrinol. Metab. Clin. N. Am..

[76] Vyzantiadis T., Theodoridou S., Giouleme O., Harsoulis P., Evgenidis N., Vyzantiadis A. (2003). Serum concentrations of insulin-like growth factor-i (igf-i) in patients with liver cirrhosis. Hepatogastroenterology.

[77] Rosario W.P. (2010). Normal values of serum igf-1 in adults: Results from a brazilian population. Arquivos Brasileiros de Endocrinologia Metabologia.

[78] Huber Y., Bierling F., Labenz C., Koch S., Schmidtmann I., Kloeckner R., Schotten S., Huber T., Lang H., Woerns M.A. (2018). Validation of insulin-like growth factor-1 as a prognostic parameter in patients with hepatocellular carcinoma in a european cohort. BMC Cancer.

[79] Cao J., Luo S.-M., Liang L., Lai J. (2007). Effects of parenteral nutrition without and with growth hormone on growth hormone/insulin-like growth factor-1 axis after hepatectomy in hepatocellular carcinoma with liver cirrhosis. J. Parenter. Enteral Nutr..

[80] Zapf J., Walter H., Froesch E.R. (1981). Radioimmunological determination of insulin-like growth factors i and ii in normal subjects and in patients with growth disorders and extrapancreatic tumour hypoglycaemia. J. Clin. Investig..

[81] Humbel R.E. (1990). Insulin-like growth factors i and ii. Eur. J. Biochem..

[82] Li X., Nadauld L., Ootani A., Corney D.C., Pai R.K., Gevaert O., Cantrell M.A., Rack P.G., Neal J.T., Chan C.W.M. (2014). Oncogenic transformation of diverse gastrointestinal tissues in primary organoid culture. Nat. Med..

[83] Martinez-Quetglas I., Pinyol R., Dauch D., Torrecilla S., Tovar V., Moeini A., Alsinet C., Portela A., Rodriguez-Carunchio L., Solé M. (2016). Igf2 is up-regulated by epigenetic mechanisms in hepatocellular carcinomas and is an actionable oncogene product in experimental models. Gastroenterology.

[84] Fumihiko H., Shin-Ichiro T. (2018). 40 years of igf1: Igf1 receptor signaling pathways. J. Mol. Endocrinol..

[85] Wang Z., Ruan Y.B., Guan Y., Liu S.H. (2003). Expression of igf-ii in early experimental hepatocellular carcinomas and its significance in early diagnosis. World J. Gastroenterol..

[86] Philippou A., Maridaki M., Pneumaticos S., Koutsilieris M. (2014). The complexity of the igf1 gene splicing, posttranslational modification and bioactivity. Mol. Med..

[87] Lara-Diaz V.J., Castilla-Cortazar I., Martín-Estal I., García-Magariño M., Aguirre G.A., Puche J.E., de la Garza R.G., Morales L.A., Muñoz U. (2017). Igf-1 modulates gene expression of proteins involved in inflammation, cytoskeleton, and liver architecture. J. Physiol. Biochem..

[88] Reiss K., Ferber A., Travali S., Porcu P., Phillips P.D., Baserga R. (1991). The protooncogene c-myb increases the expression of insulin-like growth factor 1 and insulin-like growth factor 1 receptor messenger rnas by a transcriptional mechanism. Cancer Res..

[89] Werner H., Sarfstein R., LeRoith D., Bruchim I. (2016). Insulin-like growth factor 1 signaling axis meets p53 genome protection pathways. Front. Oncol..

[90] Zhang L., Shi H., Chen H., Gong A., Liu Y., Song L., Xu X., You T., Fan X., Wang D. (2019). Dedifferentiation process driven by radiotherapy-induced hmgb1/tlr2/yap/hif-1α signaling enhances pancreatic cancer stemness. Cell Death Dis..

[91] Riedemann J., Macaulay V.M. (2006). Igf1r signalling and its inhibition. Endocr. Relat. Cancer.

[92] Vivian Hwa Y.O., Ron G. (1999). Rosenfeld The insulin-like growth factor-binding protein (igfbp) superfamily. Endocr. Rev..

[93] Clemmons D.R., Busby W.H., Arai T., Nam T.J., Clarke J.B., Jones J.I., Ankrapp D.K. (1998). Role of insulin-like growth factor binding proteins in the control of igf actions. Mol. Cell Endocrinol..

[94] Yu H., Mistry J., Nicar M.J., Khosravi M.J., Diamandis A., van Doorn J., Juul A. (1999). Insulin-like growth factors (igf-i, free igf-i, and igf-ii) and insulin-like growth factor binding proteins (igfbp-2, igfbp-3, igfbp-6, and als) in blood circulation. J. Clin. Lab. Anal..

[95] Abdelhakeem A., Kaseb A.O., Hatia R., Abdel-Wahab R., Amin H.M., Hassan M. (2019). Distribution of insulin growth factor-1 (igf-1) binding proteins in hepatocellular carcinoma with and without cirrhosis. J. Clin. Oncol..

[96] Yumoto E., Nakatsukasa H., Hanafusa T., Yumoto Y., Nouso K., Matsumoto E., Onishi T., Takuma Y., Tanaka H., Fujikawa T. (2005). Igfbp-3 expression in hepatocellular carcinoma involves abnormalities in tgf-beta and/or rb signaling pathways. Int. J. Oncol..

[97] Regel I., Eichenmüller M., Joppien S., Liebl J., Häberle B., Müller-Höcker J., Vollmar A., von Schweinitz D., Kappler R. (2012). Igfbp3 impedes aggressive growth of pediatric liver cancer and is epigenetically silenced in vascular invasive and metastatic tumors. Mol. Cancer.

[98] Jin L., Shen F., Weinfeld M., Sergi C. (2020). Insulin growth factor binding protein 7 (igfbp7)-related cancer and igfbp3 and igfbp7 crosstalk. Front. Oncol..

[99] Akiel M., Guo C., Li X., Rajasekaran D., Mendoza R.G., Robertson C.L., Jariwala N., Yuan F., Subler M.A., Windle J. (2017). Igfbp7 deletion promotes hepatocellular carcinoma. Cancer Res..

[100] Fürstenberger G., Senn H.J. (2002). Insulin-like growth factors and cancer. Lancet Oncol..

[101] Yan J., Yang X., Li L., Liu P., Wu H., Liu Z., Li Q., Liao G., Wang X. (2017). Low expression levels of insulin-like growth factor binding protein-3 are correlated with poor prognosis for patients with hepatocellular carcinoma. Oncol. Lett..

[102] Guix M., Faber A.C., Wang S.E., Olivares M.G., Song Y., Qu S., Rinehart C., Seidel B., Yee D., Arteaga C.L. (2008). Acquired resistance to egfr tyrosine kinase inhibitors in cancer cells is mediated by loss of igf-binding proteins. J. Clin. Investig..

[103] Butt A.J., Firth S.M., King M.A., Baxter R.C. (2000). Insulin-like growth factor-binding protein-3 modulates expression of bax and bcl-2 and potentiates p53-independent radiation-induced apoptosis in human breast cancer cells. J. Biol. Chem..

[104] Aleem E., Elshayeb A., Elhabachi N., Mansour A.R., Gowily A., Hela A. (2012). Serum igfbp-3 is a more effective predictor than igf-1 and igf-2 for the development of hepatocellular carcinoma in patients with chronic hcv infection. Oncol. Lett..

[105] El Tayebi H.M., Waly A.A., Assal R.A., Hosny K.A., Esmat G., Abdelaziz A.I. (2015). Transcriptional activation of the igf-ii/igf-1r axis and inhibition of igfbp-3 by mir-155 in hepatocellular carcinoma. Oncol. Lett..

[106] Subramaniam K., Ooi L.L.P.J., Hui K.M. (2010). Transcriptional down-regulation of igfbp-3 in human hepatocellular carcinoma cells is mediated by the binding of tia-1 to its at-rich element in the 3′-untranslated region. Cancer Lett..

[107] Chen D., Yoo B.K., Santhekadur P.K., Gredler R., Bhutia S.K., Das S.K., Fuller C., Su Z.Z., Fisher P.B., Sarkar D. (2011). Insulin-like growth factor-binding protein-7 functions as a potential tumor suppressor in hepatocellular carcinoma. Clin. Cancer Res..

[108] Scharf J.G., Dombrowski F., Ramadori G. (2001). The igf axis and hepatocarcinogenesis. Mol. Pathol..

[109] Tumminello F.M., Leto G., Pizzolanti G., Candiloro V., Crescimanno M., Crosta L., Flandina C., Montalto G., Soresi M., Carroccio A. (1996). Cathepsin d, b and l circulating levels as prognostic markers of malignant progression. Anticancer Res..

[110] Rajah R., Katz L., Nunn S., Solberg P., Beers T., Cohen P. (1995). Insulin-like growth factor binding protein (igfbp) proteases: Functional regulators of cell growth. Prog. Growth Factor Res..

[111] Ryan A.J., Napoletano S., Fitzpatrick P.A., Currid C.A., O’Sullivan N.C., Harmey J.H. (2009). Expression of a protease-resistant insulin-like growth factor-binding protein-4 inhibits tumour growth in a murine model of breast cancer. Br. J. Cancer.

[112] Zhang M., Smith E.P., Kuroda H., Banach W., Chernausek S.D., Fagin J.A. (2002). Targeted expression of a protease-resistant igfbp-4 mutant in smooth muscle of transgenic mice results in igfbp-4 stabilization and smooth muscle hypotrophy. J. Biol. Chem..

[113] Brahmkhatri V.P., Prasanna C., Atreya H.S. (2015). Insulin-like growth factor system in cancer: Novel targeted therapies. Biomed. Res. Int..

[114] Bunn R.C., Fowlkes J.L. (2003). Insulin-like growth factor binding protein proteolysis. Trends Endocrinol. Metab..

[115] Bach L.A., Fu P., Yang Z. (2012). Insulin-like growth factor-binding protein-6 and cancer. Clin. Sci..

[116] Tailor P.D., Kodeboyina K.S., Bai S., Patel N., Sharma S., Ratnani A., Copland J.A., She J.X., Sharma A. (2018). Diagnostic and prognostic biomarker potential of kallikrein family genes in different cancer types. Oncotarget.

[117] Brouillet J.P., Hanslick B., Maudelonde T., Pivat M.T., Grenier J., Blanc F., Rochefort H. (1991). Increased plasma cathepsin d concentration in hepatic carcinoma and cirrhosis but not in breast cancer. Clin. Biochem..

[118] Yeh H.C., Lin S.M., Chen M.F., Pan T.L., Wang P.W., Yeh C.T. (2010). Evaluation of serum matrix metalloproteinase (mmp)-9 to mmp-2 ratio as a biomarker in hepatocellular carcinoma. Hepatogastroenterology.

[119] Kim S.O., Park J.G., Lee Y.I. (1996). Increased expression of the insulin-like growth factor i (igf-i) receptor gene in hepatocellular carcinoma cell lines: Implications of igf-i receptor gene activation by hepatitis b virus x gene product. Cancer Res..

[120] Lee Y.I., Lee S., Lee Y., Bong Y.S., Hyun S.W., Yoo Y.D., Kim S.J., Kim Y.W., Poo H.R. (1998). The human hepatitis b virus transactivator x gene product regulates sp1 mediated transcription of an insulin-like growth factor ii promoter 4. Oncogene.

[121] Sohda T., Kamimura S., Iwata K., Shijo H., Okumura M. (1997). Immunohistochemical evidence of insulin-like growth factor ii in human small hepatocellular carcinoma with hepatitis c virus infection: Relationship to fatty change in carcinoma cells. J. Gastroenterol. Hepatol..

[122] Leonardi G.C., Candido S., Cervello M., Nicolosi D., Raiti F., Travali S., Spandidos D.A., Libra M. (2012). The tumor microenvironment in hepatocellular carcinoma (review). Int. J. Oncol..

[123] Liu Q., Xu Z., Mao S., Chen W., Zeng R., Zhou S., Liu J. (2015). Effect of hypoxia on hypoxia inducible factor-1α, insulin-like growth factor i and vascular endothelial growth factor expression in hepatocellular carcinoma hepg2 cells. Oncol. Lett..

[124] Xie H., Song J., Liu K., Ji H., Shen H., Hu S., Yang G., Du Y., Zou X., Jin H. (2008). The expression of hypoxia-inducible factor-1α in hepatitis b virus-related hepatocellular carcinoma: Correlation with patients’ prognosis and hepatitis b virus x protein. Dig. Dis. Sci..

[125] Yulyana Y., Ho I.A.W., Sia K.C., Newman J.P., Toh X.Y., Endaya B.B., Chan J.K.Y., Gnecchi M., Huynh H., Chung A.Y.F. (2015). Paracrine factors of human fetal mscs inhibit liver cancer growth through reduced activation of igf-1r/pi3k/akt signaling. Mol. Ther..

[126] Chen W.J., Ho C.C., Chang Y.L., Chen H.Y., Lin C.A., Ling T.Y., Yu S.L., Yuan S.S., Chen Y.J., Lin C.Y. (2014). Cancer-associated fibroblasts regulate the plasticity of lung cancer stemness via paracrine signalling. Nat. Commun..

[127] Rupp C., Scherzer M., Rudisch A., Unger C., Haslinger C., Schweifer N., Artaker M., Nivarthi H., Moriggl R., Hengstschläger M. (2015). Igfbp7, a novel tumor stroma marker, with growth-promoting effects in colon cancer through a paracrine tumor–stroma interaction. Oncogene.

[128] Sarfstein R., Werner H. (2013). Minireview: Nuclear insulin and insulin-like growth factor-1 receptors: A novel paradigm in signal transduction. Endocrinology.

[129] Aleksic T., Chitnis M.M., Perestenko O.V., Gao S., Thomas P.H., Turner G.D., Protheroe A.S., Howarth M., Macaulay V.M. (2010). Type 1 insulin-like growth factor receptor translocates to the nucleus of human tumor cells. Cancer Res..

[130] Wang Y., He L., Du Y., Zhu P., Huang G., Luo J., Yan X., Ye B., Li C., Xia P. (2015). The long noncoding rna lnctcf7 promotes self-renewal of human liver cancer stem cells through activation of wnt signaling. Cell Stem Cell.

[131] Jamwal G., Singh G., Dar M.S., Singh P., Bano N., Syed S.H., Sandhu P., Akhter Y., Monga S.P., Dar M.J. (2018). Identification of a unique loss-of-function mutation in igf1r and a crosstalk between igf1r and wnt/beta-catenin signaling pathways. Biochim. Biophys. Acta Mol. Cell Res..

[132] Bodzin A.S., Wei Z., Hurtt R., Gu T., Doria C. (2012). Gefitinib resistance in hcc mahlavu cells: Upregulation of cd133 expression, activation of igf-1r signaling pathway, and enhancement of igf-1r nuclear translocation. J. Cell Physiol..

[133] Shan J., Shen J., Liu L., Xia F., Xu C., Duan G., Xu Y., Ma Q., Yang Z., Zhang Q. (2012). Nanog regulates self-renewal of cancer stem cells through the insulin-like growth factor pathway in human hepatocellular carcinoma. Hepatology.

[134] Chang T.S., Wu Y.C., Chi C.C., Su W.C., Chang P.J., Lee K.F., Tung T.H., Wang J., Liu J.J., Tung S.Y. (2015). Activation of il6/igfir confers poor prognosis of hbv-related hepatocellular carcinoma through induction of oct4/nanog expression. Clin. Cancer Res..

[135] Tang J.J.H., Thng D.K.H., Lim J.J., Toh T.B. (2020). Jak/stat signaling in hepatocellular carcinoma. Hepat. Oncol..

[136] Verhoeven Y., Tilborghs S., Jacobs J., De Waele J., Quatannens D., Deben C., Prenen H., Pauwels P., Trinh X.B., Wouters A. (2020). The potential and controversy of targeting stat family members in cancer. Semin. Cancer Biol..

[137] Mao J., Yang H., Cui T., Pan P., Kabir N., Chen D., Ma J., Chen X., Chen Y., Yang Y. (2018). Combined treatment with sorafenib and silibinin synergistically targets both hcc cells and cancer stem cells by enhanced inhibition of the phosphorylation of stat3/erk/akt. Eur. J. Pharmacol..

[138] Chang T.S., Chen C.L., Wu Y.C., Liu J.J., Kuo Y.C., Lee K.F., Lin S.Y., Lin S.E., Tung S.Y., Kuo L.M. (2016). Inflammation promotes expression of stemness-related properties in hbv-related hepatocellular carcinoma. PLoS ONE.

[139] Lai S.C., Su Y.T., Chi C.C., Kuo Y.C., Lee K.F., Wu Y.C., Lan P.C., Yang M.H., Chang T.S., Huang Y.A.-O. (2019). Dnmt3b/oct4 expression confers sorafenib resistance and poor prognosis of hepatocellular carcinoma through il-6/stat3 regulation. J. Exp. Clin. Cancer Res..

[140] Youness R.A., El-Tayebi H.M., Assal R.A., Hosny K., Esmat G., Abdelaziz A.I. (2016). Microrna-486-5p enhances hepatocellular carcinoma tumor suppression through repression of igf-1r and its downstream mtor, stat3 and c-myc. Oncol. Lett..

[141] Zhao C., Wang Q., Wang B., Sun Q., He Z., Hong J., Kuehn F., Liu E., Zhang Z. (2017). Igf-1 induces the epithelial-mesenchymal transition via stat5 in hepatocellular carcinoma. Oncotarget.

[142] Liu L., Liu C., Zhang Q., Shen J., Zhang H., Shan J., Duan G., Guo D., Chen X., Cheng J. (2016). Sirt1-mediated transcriptional regulation of sox2 is important for self-renewal of liver cancer stem cells. Hepatology.

[143] You Y., Zheng Q., Dong Y., Xie X., Wang Y., Wu S., Zhang L., Wang Y., Xue T., Wang Z. (2016). Matrix stiffness-mediated effects on stemness characteristics occurring in hcc cells. Oncotarget.

[144] Takeda K., Mizushima T., Yokoyama Y., Hirose H., Wu X., Qian Y., Ikehata K., Miyoshi N., Takahashi H., Haraguchi N. (2018). Sox2 is associated with cancer stem-like properties in colorectal cancer. Sci. Rep..

[145] Apostolou P., Toloudi M., Chatziioannou M., Ioannou E., Papasotiriou I. (2012). Cancer stem cells stemness transcription factors expression correlates with breast cancer disease stage. Curr. Stem Cell Res. Ther..

[146] Müller M., Hermann P.C., Liebau S., Weidgang C., Seufferlein T., Kleger A., Perkhofer L. (2016). The role of pluripotency factors to drive stemness in gastrointestinal cancer. Stem Cell Res..

[147] Quan M.Y., Guo Q., Liu J., Yang R., Bai J., Wang W., Cai Y., Han R., Lv Y.Q., Ding L. (2020). An fgfr/akt/sox2 signaling axis controls pancreatic cancer stemness. Front. Cell Dev. Biol..

[148] Bu Y., Jia Q.A., Ren Z.G., Zhang J.B., Jiang X.M., Liang L., Xue T.C., Zhang Q.B., Wang Y.H., Zhang L. (2014). Maintenance of stemness in oxaliplatin-resistant hepatocellular carcinoma is associated with increased autocrine of igf1. PLoS ONE.

[149] Xia Q., Han T., Yang P., Wang R., Li H., Zhang J., Zhou X. (2019). Microrna-28-5p regulates liver cancer stem cell expansion via igf-1 pathway. Stem Cells Int..

[150] Chen P.C., Kuo Y.C., Chuong C.M., Huang Y.H. (2021). Niche modulation of igf-1r signaling: Its role in stem cell pluripotency, cancer reprogramming, and therapeutic applications. Front. Cell Dev. Biol..

[151] Halder G., Johnson R.L. (2011). Hippo signaling: Growth control and beyond. Development.

[152] Zhao B., Li L., Lei Q., Guan K.L. (2010). The hippo-yap pathway in organ size control and tumorigenesis: An updated version. Genes Dev..

[153] Zhang T., Zhou Q., Pignoni F. (2011). Yki/yap, sd/tead and hth/meis control tissue specification in the drosophila eye disc epithelium. PLoS ONE.

[154] Chan S.W., Lim C.J., Loo L.S., Chong Y.F., Huang C., Hong W. (2009). Teads mediate nuclear retention of taz to promote oncogenic transformation. J. Biol. Chem..

[155] Mahoney W.M., Hong J.-H., Yaffe M.B., Farrance I.K.G. (2005). The transcriptional co-activator taz interacts differentially with transcriptional enhancer factor-1 (tef-1) family members. Biochem. J..

[156] Vassilev A., Kaneko K.J., Shu H., Zhao Y., DePamphilis M.L. (2001). Tead/tef transcription factors utilize the activation domain of yap65, a src/yes-associated protein localized in the cytoplasm. Genes Dev..

[157] Zhao B., Ye X., Yu J., Li L., Li W., Li S., Yu J., Lin J.D., Wang C.Y., Chinnaiyan A.M. (2008). Tead mediates yap-dependent gene induction and growth control. Genes Dev..

[158] Cui C.B., Cooper L.F., Yang X., Karsenty G., Aukhil I. (2003). Transcriptional coactivation of bone-specific transcription factor cbfa1 by taz. Mol. Cell Biol..

[159] Qiao Y., Lin S.J., Chen Y., Voon D.-c., Zhu F., Chuang L.S.H., Wang T., Tan P., Lee S.C., Yeoh K.G. (2015). Runx3 is a novel negative regulator of oncogenic TEAD-YAP complex in gastric cancer. Oncogene.

[160] Murakami M., Nakagawa M., Olson E.N., Nakagawa O. (2005). A ww domain protein taz is a critical coactivator for tbx5, a transcription factor implicated in Holt-Oram syndrome. Proc. Natl. Acad. Sci. USA.

[161] Strassburger K., Tiebe M., Pinna F., Breuhahn K., Teleman A.A. (2012). Insulin/igf signaling drives cell proliferation in part via yorkie/yap. Dev. Biol..

[162] Borreguero-Muñoz N., Fletcher G.C., Aguilar-Aragon M., Elbediwy A., Vincent-Mistiaen Z.I., Thompson B.J. (2019). The hippo pathway integrates pi3k-akt signals with mechanical and polarity cues to control tissue growth. PLoS Biol..

[163] Zhu H., Wang D.D., Yuan T., Yan F.J., Zeng C.M., Dai X.Y., Chen Z.b., Chen Y., Zhou T., Fan G.H. (2018). Multikinase inhibitor ct-707 targets liver cancer by interrupting the hypoxia-activated igf-1r–yap axis. Cancer Res..

[164] Chang H.L., Chen H.A., Bamodu O.A., Lee K.F., Tzeng Y.M., Lee W.H., Tsai J.T. (2018). Ovatodiolide suppresses yes-associated protein 1-modulated cancer stem cell phenotypes in highly malignant hepatocellular carcinoma and sensitizes cancer cells to chemotherapy in vitro. Toxicol. In Vitro.

[165] Van Haele M., Moya I.M., Karaman R., Rens G., Snoeck J., Govaere O., Nevens F., Verslype C., Topal B., Monbaliu D. (2019). Yap and taz heterogeneity in primary liver cancer: An analysis of its prognostic and diagnostic role. Int. J. Mol. Sci..

[166] Zhu P., Wang Y., Wu J., Huang G., Liu B., Ye B., Du Y., Gao G., Tian Y., He L. (2016). Lncbrm initiates yap1 signalling activation to drive self-renewal of liver cancer stem cells. Nat. Commun..

[167] Yimlamai D., Christodoulou C., Galli G.G., Yanger K., Pepe-Mooney B., Gurung B., Shrestha K., Cahan P., Stanger B.Z., Camargo F.D. (2014). Hippo pathway activity influences liver cell fate. Cell.

[168] Kim G.J., Kim H., Park Y.N. (2013). Increased expression of yes-associated protein 1 in hepatocellular carcinoma with stemness and combined hepatocellular-cholangiocarcinoma. PLoS ONE.

[169] Qiu L.G., Li H.L., Fu S.R., Chen X.F., Lu L.G. (2018). Surface markers of liver cancer stem cells and innovative targeted-therapy strategies for hcc (review). Oncol. Lett..

[170] Sun J.H., Luo Q., Liu L.L., Song G.B. (2016). Liver cancer stem cell markers: Progression and therapeutic implications. World J. Gastroenterol..

[171] Schulte L.A., Lopez-Gil J.C., Sainz B., Hermann P.C. (2020). The cancer stem cell in hepatocellular carcinoma. Cancers.

[172] Benabou E., Salame Z., Wendum D., Lequoy M., Tahraoui S., Merabtene F., Chretien Y., Scatton O., Rosmorduc O., Fouassier L. (2019). Insulin receptor isoform a favors tumor progression in human hepatocellular carcinoma by increasing stem/progenitor cell features. Cancer Lett..

[173] Ye Y., Guo J., Xiao P., Ning J., Zhang R., Liu P., Yu W., Xu L., Zhao Y., Yu J. (2020). Macrophages-induced long noncoding rna h19 up-regulation triggers and activates the mir-193b/mapk1 axis and promotes cell aggressiveness in hepatocellular carcinoma. Cancer Lett..

[174] Zheng X., Li C., Yu K., Shi S., Chen H., Qian Y., Mei Z. (2020). Aquaporin-9, mediated by igf2, suppresses liver cancer stem cell properties via augmenting ros/beta-catenin/foxo3a signaling. Mol. Cancer Res..

[175] He J., Xiong L., Li Q., Lin L., Miao X., Yan S., Hong Z., Yang L., Wen Y., Deng X. (2017). 3d modeling of cancer stem cell niche. Oncotarget.

[176] Borovski T., De Sousa E.M.F., Vermeulen L., Medema J.P. (2011). Cancer stem cell niche: The place to be. Cancer Res..

[177] Plaks V., Kong N., Werb Z. (2015). The cancer stem cell niche: How essential is the niche in regulating stemness of tumor cells?. Cell Stem Cell.

[178] Ye J., Wu D., Wu P., Chen Z., Huang J. (2014). The cancer stem cell niche: Cross talk between cancer stem cells and their microenvironment. Tumor Biol..

[179] Melzer C., von der Ohe J., Lehnert H., Ungefroren H., Hass R. (2017). Cancer stem cell niche models and contribution by mesenchymal stroma/stem cells. Mol. Cancer.

[180] Bendall S.C., Stewart M.H., Menendez P., George D., Vijayaragavan K., Werbowetski-Ogilvie T., Ramos-Mejia V., Rouleau A., Yang J., Bossé M. (2007). Igf and fgf cooperatively establish the regulatory stem cell niche of pluripotent human cells in vitro. Nature.

[181] Youssef A., Aboalola D., Han V.K. (2017). The roles of insulin-like growth factors in mesenchymal stem cell niche. Stem Cells Int..

[182] Sanchez-Lopez E., Flashner-Abramson E., Shalapour S., Zhong Z., Taniguchi K., Levitzki A., Karin M. (2016). Targeting colorectal cancer via its microenvironment by inhibiting igf-1 receptor-insulin receptor substrate and stat3 signaling. Oncogene.

[183] Chan T.S., Shaked Y., Tsai K.K. (2019). Targeting the interplay between cancer fibroblasts, mesenchymal stem cells, and cancer stem cells in desmoplastic cancers. Front. Oncol..

[184] Beasley R.P., Hwang L.Y., Lin C.C., Chien C.S. (1981). Hepatocellular carcinoma and hepatitis b virus. A prospective study of 22 707 men in taiwan. Lancet.

[185] Ng K.Y., Chai S., Tong M., Guan X.Y., Lin C.H., Ching Y.P., Xie D., Cheng A.S., Ma S. (2016). C-terminal truncated hepatitis b virus x protein promotes hepatocellular carcinogenesis through induction of cancer and stem cell-like properties. Oncotarget.

[186] Torresi J., Tran B.M., Christiansen D., Earnest-Silveira L., Schwab R.H.M., Vincan E. (2019). Hbv-related hepatocarcinogenesis: The role of signalling pathways and innovative ex vivo research models. BMC Cancer.

[187] D’Arville C.N., Nouri-Aria K.T., Johnson P., Williams R. (1991). Regulation of insulin-like growth factor ii gene expression by hepatitis b virus in hepaocellular carcinoma. Hepatology.

[188] Ji Y., Wang Z., Chen H., Zhang L., Zhuo F., Yang Q. (2018). Serum from chronic hepatitis b patients promotes growth and proliferation via the igf-ii/igf-ir/mek/erk signaling pathway in hepatocellular carcinoma cells. Cell Physiol. Biochem..

[189] Nielsen K.O., Mirza A.H., Kaur S., Jacobsen K.S., Winther T.N., Glebe D., Pociot F., Hogh B., Størling J. (2018). Hepatitis b virus suppresses the secretion of insulin-like growth factor binding protein 1 to facilitate anti-apoptotic igf-1 effects in hepg2 cells. Exp. Cell Res..

[190] Mani S.K., Zhang H., Diab A., Pascuzzi P.E., Lefrancois L., Fares N., Bancel B., Merle P., Andrisani O. (2016). Epcam-regulated intramembrane proteolysis induces a cancer stem cell-like gene signature in hepatitis b virus-infected hepatocytes. J. Hepatol..

[191] Ching R.H.H., Sze K.M.F., Lau E.Y.T., Chiu Y.T., Lee J.M.F., Ng I.O.L., Lee T.K.W. (2017). C-terminal truncated hepatitis b virus x protein regulates tumorigenicity, self-renewal and drug resistance via stat3/nanog signaling pathway. Oncotarget.

[192] Korkaya H., Liu S., Wicha M.S. (2011). Regulation of cancer stem cells by cytokine networks: Attacking cancer’s inflammatory roots. Clin. Cancer Res..

[193] Zhang S., Yang X., Wang L., Zhang C. (2018). Interplay between inflammatory tumor microenvironment and cancer stem cells. Oncol. Lett..

[194] Wang X., Sun W., Shen W., Xia M., Chen C., Xiang D., Ning B., Cui X., Li H., Li X. (2016). Long non-coding rna dilc regulates liver cancer stem cells via il-6/stat3 axis. J. Hepatol..

[195] Zhao B., Wang Y., Tan X., Ke K., Zheng X., Wang F., Lan S., Liao N., Cai Z., Shi Y. (2019). Inflammatory micro-environment contributes to stemness properties and metastatic potential of hcc via the nf-kappab/mir-497/sall4 axis. Mol. Ther. Oncolytics.

[196] Liu S., Tan W.Y., Chen Q.R., Chen X.P., Fu K., Zhao Y.Y., Chen Z.W. (2008). Daintain/aif-1 promotes breast cancer proliferation via activation of the nf-kappab/cyclin d1 pathway and facilitates tumor growth. Cancer Sci..

[197] Li T., Feng Z., Jia S., Wang W., Du Z., Chen N., Chen Z. (2012). Daintain/aif-1 promotes breast cancer cell migration by up-regulated tnf-alpha via activate p38 mapk signaling pathway. Breast Cancer Res. Treat..

[198] Elizondo D.M., Brandy N.Z.D., da Silva R.L.L., Haddock N.L., Kacsinta A.D., de Moura T.R., Lipscomb M.W. (2019). Allograft inflammatory factor-1 governs hematopoietic stem cell differentiation into cdc1 and monocyte-derived dendritic cells through irf8 and relb in vitro. Front. Immunol..

[199] Jia S., Du Z., Jiang H., Huang X., Chen Z., Chen N. (2015). Daintain/aif-1 accelerates the activation of insulin-like growth factor-1 receptor signaling pathway in hepg2 cells. Oncol. Rep..

[200] Guo Q., Yu D.-Y., Yang Z.-F., Liu D.-Y., Cao H.-Q., Liao X.-W. (2020). Igfbp2 upregulates zeb1 expression and promotes hepatocellular carcinoma progression through nf-κb signaling pathway. Dig. Liver Dis..

[201] Liu F., Sun Y., Liu B., Lu J., Li H., Zhu H., Gao H., Zhou X., Chang H. (2018). Insulin-like growth factor-1 induces epithelial-mesenchymal transition in hepatocellular carcinoma by activating survivin. Oncol. Rep..

[202] Adnane L., Trail P.A., Taylor I., Wilhelm S.M. (2006). Sorafenib (bay 43-9006, nexavar®), a dual-action inhibitor that targets raf/mek/erk pathway in tumor cells and tyrosine kinases vegfr/pdgfr in tumor vasculature. Methods Enzymol..

[203] Keating G.M., Santoro A. (2009). Sorafenib. Drugs.

[204] Llovet J.M., Bruix J. (2008). Molecular targeted therapies in hepatocellular carcinoma. Hepatology.

[205] Costa R., Carneiro B.A., Chandra S., Pai S.G., Chae Y.K., Kaplan J.B., Garrett H.B., Agulnik M., Kopp P.A., Giles F.J. (2016). Spotlight on lenvatinib in the treatment of thyroid cancer: Patient selection and perspectives. Drug Des. Devel. Ther..

[206] Zschäbitz S., Grüllich C. (2018). Lenvantinib: A tyrosine kinase inhibitor of vegfr 1-3, fgfr 1-4, pdgfrα, kit and ret. Recent Results Cancer Res..

[207] Nakazawa Y., Kawano S., Matsui J., Funahashi Y., Tohyama O., Muto H., Nakagawa T., Matsushima T. (2015). Multitargeting strategy using lenvatinib and golvatinib: Maximizing anti-angiogenesis activity in a preclinical cancer model. Cancer Sci..

[208] Bruix J., Qin S., Merle P., Granito A., Huang Y.H., Bodoky G., Pracht M., Yokosuka O., Rosmorduc O., Breder V. (2017). Regorafenib for patients with hepatocellular carcinoma who progressed on sorafenib treatment (resorce): A randomised, double-blind, placebo-controlled, phase 3 trial. Lancet.

[209] Duensing A., Boichuk S., Rausch J. (2013). New developments in management of gastrointestinal stromal tumors: Regorafenib, the new player in the team. Gastrointest. Cancer Targets Ther..

[210] Tai W.T., Chu P.Y., Shiau C.W., Chen Y.L., Li Y.S., Hung M.H., Chen L.J., Chen P.L., Su J.C., Lin P.Y. (2014). Stat3 mediates regorafenib-induced apoptosis in hepatocellular carcinoma. Clin. Cancer Res..

[211] Fondevila F., Méndez-Blanco C., Fernández-Palanca P., González-Gallego J., Mauriz J.L. (2019). Anti-tumoral activity of single and combined regorafenib treatments in preclinical models of liver and gastrointestinal cancers. Exp. Mol. Med..

[212] Finn R.S. (2017). Review of regorafenib for the treatment of hepatocellular carcinoma. Gastroenterol. Hepatol..

[213] Deeks E.D. (2019). Cabozantinib: A review in advanced hepatocellular carcinoma. Target. Oncol..

[214] Trojan J. (2020). Cabozantinib for the treatment of advanced hepatocellular carcinoma: Current data and future perspectives. Drugs.

[215] Abou-Alfa G.K., Meyer T., Cheng A.L., El-Khoueiry A.B., Rimassa L., Ryoo B.Y., Cicin I., Merle P., Chen Y., Park J.W. (2018). Cabozantinib in patients with advanced and progressing hepatocellular carcinoma. N. Engl. J. Med..

[216] De Luca E., Marino D., Di Maio M. (2020). Ramucirumab, a second-line option for patients with hepatocellular carcinoma: A review of the evidence. Cancer Manag. Res..

[217] Zhu A.X., Kang Y.-K., Yen C.-J., Finn R.S., Galle P.R., Llovet J.M., Assenat E., Brandi G., Pracht M., Lim H.Y. (2019). Ramucirumab after sorafenib in patients with advanced hepatocellular carcinoma and increased α-fetoprotein concentrations (reach-2): A randomised, double-blind, placebo-controlled, phase 3 trial. Lancet Oncol..

[218] Yen C.J., Kudo M., Lim H.Y., Hsu C.H., Vogel A., Brandi G., Cheng R., Nitu I.S., Abada P., Hsu Y. (2020). Efficacy and safety of ramucirumab in asian and non-asian patients with advanced hepatocellular carcinoma and elevated alpha-fetoprotein: Pooled individual data analysis of two randomized studies. Liver Cancer.

[219] Kuzuya T., Ishigami M., Ito T., Ishizu Y., Honda T., Ishikawa T., Fujishiro M. (2020). Initial experience of ramucirumab treatment after lenvatinib failure for patients with advanced hepatocellular carcinoma. Anticancer Res..

[220] El-Khoueiry A.B., Sangro B., Yau T., Crocenzi T.S., Kudo M., Hsu C., Kim T.-Y., Choo S.-P., Trojan J., Welling T.H. (2017). Nivolumab in patients with advanced hepatocellular carcinoma (checkmate 040): An open-label, non-comparative, phase 1/2 dose escalation and expansion trial. Lancet.

[221] Yau T., Park J.W., Finn R.S., Cheng A.L., Mathurin P., Edeline J., Kudo M., Han K.H., Harding J.J., Merle P. (2019). Lba38_pr - checkmate 459: A randomized, multi-center phase iii study of nivolumab (nivo) vs sorafenib (sor) as first-line (1l) treatment in patients (pts) with advanced hepatocellular carcinoma (ahcc). Ann. Oncol..

[222] Zhu A.X., Finn R.S., Edeline J., Cattan S., Ogasawara S., Palmer D., Verslype C., Zagonel V., Fartoux L., Vogel A. (2018). Pembrolizumab in patients with advanced hepatocellular carcinoma previously treated with sorafenib (keynote-224): A non-randomised, open-label phase 2 trial. Lancet Oncol..

[223] Finn R.S., Ryoo B.Y., Merle P., Kudo M., Bouattour M., Lim H.Y., Breder V., Edeline J., Chao Y., Ogasawara S. (2020). Pembrolizumab as second-line therapy in patients with advanced hepatocellular carcinoma in keynote-240: A randomized, double-blind, phase iii trial. J. Clin. Oncol..

[224] Llovet J.M., Zucman-Rossi J., Pikarsky E., Sangro B., Schwartz M., Sherman M., Gores G. (2016). Hepatocellular carcinoma. Nat. Rev. Dis. Primers.

[225] Colagrande S., Inghilesi A.L., Aburas S., Taliani G.G., Nardi C., Marra F. (2016). Challenges of advanced hepatocellular carcinoma. World J. Gastroenterol..

[226] Llovet J.M., Hernandez-Gea V. (2014). Hepatocellular carcinoma: Reasons for phase iii failure and novel perspectives on trial design. Clin. Cancer Res..

[227] Tang W., Chen Z., Zhang W., Cheng Y., Zhang B., Wu F., Wang Q., Wang S., Rong D., Reiter F.P. (2020). The mechanisms of sorafenib resistance in hepatocellular carcinoma: Theoretical basis and therapeutic aspects. Signal. Transduct. Target. Ther..

[228] Kim C.M., Hwang S., Keam B., Yu Y.S., Kim J.H., Kim D.S., Bae S.H., Kim G.D., Lee J.K., Seo Y.B. (2020). Gene signature for sorafenib susceptibility in hepatocellular carcinoma: Different approach with a predictive biomarker. Liver Cancer.

[229] Lin X., Li A.-m., Li Y.-H., Luo R.-C., Zou Y.-J., Liu Y.-Y., Liu C., Xie Y.-Y., Zuo S., Liu Z. (2020). Silencing myh9 blocks hbx-induced gsk3β ubiquitination and degradation to inhibit tumor stemness in hepatocellular carcinoma. Signal. Transduct. Target. Ther..

[230] Witt-Kehati D., Fridkin A., Alaluf M.B., Zemel R., Shlomai A. (2018). Inhibition of pmapk14 overcomes resistance to sorafenib in hepatoma cells with hepatitis b virus. Transl. Oncol..

[231] Lu S.-N., Wang J.-H., Su C.-W., Wang T.-E., Dai C.-Y., Chen C.-H., Chen R.-C., Yang S.-S., Hung C.-F., Huang S.-F. (2018). Management consensus guideline for hepatocellular carcinoma: 2016 updated by the Taiwan liver cancer association and the gastroenterological society of Taiwan. J. Formosan Med. Assoc..

[232] Wang F., Bank T., Malnassy G., Arteaga M., Shang N., Dalheim A., Ding X., Cotler S.J., Denning M.F., Nishimura M.I. (2018). Inhibition of insulin-like growth factor 1 receptor enhances the efficacy of sorafenib in inhibiting hepatocellular carcinoma cell growth and survival. Hepatol. Commun..

[233] Jeannot V., Busser B., Vanwonterghem L., Michallet S., Ferroudj S., Cokol M., Coll J.L., Ozturk M., Hurbin A. (2016). Synergistic activity of vorinostat combined with gefitinib but not with sorafenib in mutant kras human non-small cell lung cancers and hepatocarcinoma. Onco Targets Ther..

[234] Tomizawa M., Shinozaki F., Motoyoshi Y., Sugiyama T., Yamamoto S., Sueishi M. (2014). Picropodophyllin and sorafenib synergistically suppress the proliferation and motility of hepatocellular carcinoma cells. Oncol. Lett..

[235] Tovar V., Cornella H., Moeini A., Vidal S., Hoshida Y., Sia D., Peix J., Cabellos L., Alsinet C., Torrecilla S. (2017). Tumour initiating cells and igf/fgf signalling contribute to sorafenib resistance in hepatocellular carcinoma. Gut.

[236] Macfarlane L.-A., Murphy P.R. (2010). Microrna: Biogenesis, function and role in cancer. Curr. Genom..

[237] Volinia S., Calin G.A., Liu C.G., Ambs S., Cimmino A., Petrocca F., Visone R., Iorio M., Roldo C., Ferracin M. (2006). A microrna expression signature of human solid tumors defines cancer gene targets. Proc. Natl. Acad. Sci. USA.

[238] Kutay H., Bai S., Datta J., Motiwala T., Pogribny I., Frankel W., Jacob S.T., Ghoshal K. (2006). Downregulation of mir-122 in the rodent and human hepatocellular carcinomas. J. Cell Biochem..

[239] Xu Y., Huang J., Ma L., Shan J., Shen J., Yang Z., Liu L., Luo Y., Yao C., Qian C. (2016). Microrna-122 confers sorafenib resistance to hepatocellular carcinoma cells by targeting igf-1r to regulate ras/raf/erk signaling pathways. Cancer Lett..

[240] Hu J., Zhang J., Sun F., Qi M., Su P., Liu H., Gao L., Jiao M., Wu Z., Xiang L. (2019). Enhancer of zeste 2 polycomb repressive complex 2 subunit promotes sorafenib resistance of hepatocellular carcinoma though insulin-like growth factor 1 receptor. Anticancer Drugs.

[241] Lin Z., Xia S., Liang Y., Ji L., Pan Y., Jiang S., Wan Z., Tao L., Chen J., Lin C. (2020). Lxr activation potentiates sorafenib sensitivity in hcc by activating microrna-378a transcription. Theranostics.

[242] Allard J.B., Duan C. (2018). Igf-binding proteins: Why do they exist and why are there so many?. Front. Endocrinol..

[243] Mazerbourg S., Monget P. (2018). Insulin-like growth factor binding proteins and igfbp proteases: A dynamic system regulating the ovarian folliculogenesis. Front. Endocrinol..

[244] Weng X., Wu J., Lv Z., Peng C., Chen J., Zhang C., He B., Tong R., Hu W., Ding C. (2019). Targeting mybbp1a suppresses hcc progression via inhibiting igf1/akt pathway by cpg islands hypo-methylation dependent promotion of igfbp5. EBioMedicine.

[245] He J., Zuo Q., Hu B., Jin H., Wang C., Cheng Z., Deng X., Yang C., Ruan H., Yu C. (2019). A novel, liver-specific long noncoding rna linc01093 suppresses hcc progression by interaction with igf2bp1 to facilitate decay of gli1 mrna. Cancer Lett..

[246] Lee Y.-Y., Mok M.T., Kang W., Yang W., Tang W., Wu F., Xu L., Yan M., Yu Z., Lee S.-D. (2018). Loss of tumor suppressor igfbp4 drives epigenetic reprogramming in hepatic carcinogenesis. Nucleic Acids Res..

[247] Gao Y., Fan X., Li N., Du C., Yang B., Qin W., Fu J., Markowitz G.J., Wang H., Ma J. (2020). Ccl22 signaling contributes to sorafenib resistance in hepatitis b virus-associated hepatocellular carcinoma. Pharmacol. Res..

[248] Saile B., DiRocco P., Dudas J., El-Armouche H., Sebb H., Eisenbach C., Neubauer K., Ramadori G. (2004). Igf-i induces DNA synthesis and apoptosis in rat liver hepatic stellate cells (hsc) but DNA synthesis and proliferation in rat liver myofibroblasts (rmf). Lab. Investig..

[249] Park S.Y., Lee Y.K., Kim H.J., Park O.J., Kim Y.M. (2016). Ampk interacts with β-catenin in the regulation of hepatocellular carcinoma cell proliferation and survival with selenium treatment. Oncol. Rep..

[250] Lippolis C., Refolo M.G., D’Alessandro R., Carella N., Messa C., Cavallini A., Carr B.I. (2015). Resistance to multikinase inhibitor actions mediated by insulin like growth factor-1. J. Exp. Clin. Cancer Res..

[251] Suemura S., Kodama T., Myojin Y., Yamada R., Shigekawa M., Hikita H., Sakamori R., Tatsumi T., Takehara T. (2019). Crispr loss-of-function screen identifies the hippo signaling pathway as the mediator of regorafenib efficacy in hepatocellular carcinoma. Cancers.

[252] Shi W., Zhang S., Ma D., Yan D., Zhang G., Cao Y., Wang Z., Wu J., Jiang C. (2020). Targeting sphk2 reverses acquired resistance of regorafenib in hepatocellular carcinoma. Front. Oncol..

[253] Wu L.W., Zhou D.M., Zhang Z.Y., Zhang J.K., Zhu H.J., Lin N.M., Zhang C. (2019). Suppression of lsd1 enhances the cytotoxic and apoptotic effects of regorafenib in hepatocellular carcinoma cells. Biochem. Biophys. Res. Commun..

[254] Liu R., Li Y., Tian L., Shi H., Wang J., Liang Y., Sun B., Wang S., Zhou M., Wu L. (2019). Gankyrin drives metabolic reprogramming to promote tumorigenesis, metastasis and drug resistance through activating β-catenin/c-myc signaling in human hepatocellular carcinoma. Cancer Lett..

[255] Refolo M.G., Lippolis C., Carella N., Cavallini A., Messa C., D’Alessandro R. (2018). Chlorogenic acid improves the regorafenib effects in human hepatocellular carcinoma cells. Int. J. Mol. Sci..

[256] Desbois-Mouthon C., Cacheux W., Blivet-Van Eggelpoel M.J., Barbu V., Fartoux L., Poupon R., Housset C., Rosmorduc O. (2006). Impact of igf-1r/egfr cross-talks on hepatoma cell sensitivity to gefitinib. Int. J. Cancer.

[257] Ezzoukhry Z., Louandre C., Trecherel E., Godin C., Chauffert B., Dupont S., Diouf M., Barbare J.C., Maziere J.C., Galmiche A. (2012). Egfr activation is a potential determinant of primary resistance of hepatocellular carcinoma cells to sorafenib. Int. J. Cancer.

[258] Fu R., Jiang S., Li J., Chen H., Zhang X. (2020). Activation of the hgf/c-met axis promotes lenvatinib resistance in hepatocellular carcinoma cells with high c-met expression. Med. Oncol..

[259] Abou-Alfa G.K., Capanu M., O’Reilly E.M., Ma J., Chou J.F., Gansukh B., Shia J., Kalin M., Katz S., Abad L. (2014). A phase ii study of cixutumumab (imc-a12, nsc742460) in advanced hepatocellular carcinoma. J. Hepatol..

[260] El-Khoueiry A.B., O’Donnell R., Semrad T.J., Mack P., Blanchard S., Bahary N., Jiang Y., Yen Y., Wright J., Chen H. (2018). A phase i trial of escalating doses of cixutumumab (imc-a12) and sorafenib in the treatment of advanced hepatocellular carcinoma. Cancer Chemother. Pharmacol..

[261] Wu J., Zhu A.X. (2011). Targeting insulin-like growth factor axis in hepatocellular carcinoma. J. Hematol. Oncol..

[262] Faivre S.J., Fartoux L., Bouattour M., Bumsel F., Dreyer C., Raymond E., Rosmorduc O. (2011). A phase i study of ave1642, a human monoclonal antibody–blocking insulin-like growth factor-1 receptor (igf-1r), given as a single agent and in combination with sorafenib as first-line therapy in patients with advanced hepatocellular carcinoma (hcc). J. Clin. Oncol..

[263] McKian K.P., Haluska P. (2009). Cixutumumab. Expert Opin. Investig. Drugs.

[264] Abou-Alfa G.K., Gansukh B., Chou J.F., Shia J., Capanu M., Kalin M., Chen H.X., Zojwalla N.J., Katz S., Reidy D.L. (2011). Phase ii study of cixutumumab (imc-a12, nsc742460; c) in hepatocellular carcinoma (hcc). J. Clin. Oncol..

[265] Prenner S., Kulik L., Sanyal A.J., Boyer T.D., Lindor K.D., Terrault N.A. (2018). 46-hepatocellular carcinoma. Zakim and Boyer’s Hepatology.

[266] Rieder S., Michalski C.W., Friess H., Kleeff J. (2011). Insulin-like growth factor signaling as a therapeutic target in pancreatic cancer. Anticancer Agents Med. Chem..

